# Genome-wide association study for performance traits in chickens using genotype by sequencing approach

**DOI:** 10.1038/srep41748

**Published:** 2017-02-09

**Authors:** Fábio Pértille, Gabriel Costa Monteiro Moreira, Ricardo Zanella, José de Ribamar da Silva Nunes, Clarissa Boschiero, Gregori Alberto Rovadoscki, Gerson Barreto Mourão, Mônica Corrêa Ledur, Luiz Lehmann Coutinho

**Affiliations:** 1 University of São Paulo (USP)/Luiz de Queiroz College of Agriculture (ESALQ), Piracicaba, São Paulo, Brazil; 2 College of Agronomy and Veterinary Medicine, Veterinary School, University of Passo Fundo, Rio Grande do Sul, Brazil; 3 Embrapa Suínos e Aves, Concórdia, Santa Catarina, Brazil

## Abstract

Performance traits are economically important and are targets for selection in breeding programs, especially in the poultry industry. To identify regions on the chicken genome associated with performance traits, different genomic approaches have been applied in the last years. The aim of this study was the application of CornellGBS approach (134,528 SNPs generated from a *PstI* restriction enzyme) on Genome-Wide Association Studies (GWAS) in an outbred F_2_ chicken population. We have validated 91.7% of these 134,528 SNPs after imputation of missed genotypes. Out of those, 20 SNPs were associated with feed conversion, one was associated with body weight at 35 days of age (P < 7.86E-07) and 93 were suggestively associated with a variety of performance traits (P < 1.57E-05). The majority of these SNPs (86.2%) overlapped with previously mapped QTL for the same performance traits and some of the SNPs also showed novel potential QTL regions. The results obtained in this study suggests future searches for candidate genes and QTL refinements as well as potential use of the SNPs described here in breeding programs.

Production efficiency in the poultry industry is constantly improving as a result of selection for growth rate, feed efficiency and carcass traits for broilers, and egg production and quality traits for layers^1^,^2^. The understanding of genomic information of loci controlling those traits are important to improvement of selection efficiencies of breeding programs^1^.

Several studies have been conducted in chickens using markers randomly distributed in the genome (microsatellites), which have allowed the identification of several QTLs for production traits^3^,^4^,^5^,^6^,^7^,^8^,^9^,^10^,^11^,^12^,^13^. Following these, some of the studies have focused their attention on the identification of SNPs in functional and positional candidate genes and to test their association on target QTL regions^14^,^15^,^16^,^17^,^18^,^19^. With the advent of next-generation sequencing (NGS), it was possible to identify a global SNP profile and to perform genome-wide association studies (GWAS) to find novel QTL regions^20^,^21^,^22^,^23^,^24^,^25^,^26^ and also to refine the previously published regions^27^,^28^,^29^,^30^,^31^.

Despite the high-throughput data generation by NGS, which have facilitated the identification of SNPs in several populations, the use of this method for GWAS is still a limitation. This is caused by the high cost involved with the generation of data to be applied in a large number of individuals. To solve this high cost problem, SNP panels were designed to be applied in GWAS^32^,^33^. However, some important regions in the genome are inaccessible to sequence capture approaches^34^ mainly because they are based on predesigned SNP profiles. To overcome those limitations, and to present a unique SNP profile, we used the *PstI*-derived SNPs dataset from CornellGBS optimized approach. This dataset was originated from a SNP call from the reduced representation of the sequenced genome (~5%) through *PstI* restriction enzyme^35^. This SNP dataset is reliable and reproducible, showing a unique profile of SNPs with microchromosome enrichment^35^ that contains 2-4 times higher gene density than macrochromosomes^36^,^37^.

The aim of this study was to identify genetic markers using *PstI*-derived SNPs dataset, and further use that information to conduct a GWAS with performance traits in chickens. In addition, we have performed a linkage disequilibrium (LD) analyses in the parental, F_1_ and F_2_ generations, to better understand the segregation of haplotype blocks, and the population structure, from the associated and suggestively associated SNPs identified. Finally, we have compared the location of these mentioned SNPs with known QTLs, with the objective to validate and to refine the regions of known QTLs.

## Results

### Animals and Phenotypes

The descriptive statistics for the eight performance traits analyzed can be observed in Table 1. Detailed description of these animals and traits were provided elsewhere^7^,^16^. The large variability is expected since the animals are from a broiler x layer F_2_ population.

### Genotypes

In our previously work^35^, using a minimum taxon call rate of 90%, we have identified 67,096 SNPs originated from 462 chickens using the GBS approach. However, in this study, different filter parameters were applied. We have reduced the loci call rate filtering criteria to 70%. This parameter is the minimum threshold of individuals call rate for each loci to be included in the output. This reduction had minimal impact on sample call rate (proportion of missing genotypes per individual) and large impact on number of SNPs. The sample call rate reduced from 99.96% ± 0.04% to 99.90% ± 0.1% and the number of SNPs increased from 67,096 to 134,528. This allowed us to capture more SNPs, but the number of missing genotypes increased (for details, see M&M section). To overcome this, we have imputed the missing genotypes using Beagle 4.1 software^38^. This approach resulted in a panel of 134,528 derived *PstI*-SNPs present in all animals.

### SNPs validation

The dataset of 134,528 SNP chromosomal positions obtained with the CornellGBS before and after the imputation analysis was compared with the 600 K Affymetrix® HD genotyping array dataset in order to perform a method validation, since both sets were obtained from the same animals (5 individuals from F_2_-7810 family). The genotype concordance of the SNPs with concordant chromosomal positions detected between the two methods is shown in Table 2. On average, 91.80%, and 91.66% of the SNPs had concordant genotypes between the CornellGBS and 600 K datasets before and after imputation, respectively. The accuracy of the heterozygous genotypes was slightly lower after the imputation. Reduced representation methods, like CornellGBS, has limitations calling the heterozygous markers^39^. In our study, we have observed that 82.14 and 82.30% of heterozygous SNPs, while 97.97 and 97.65% of all homozygous SNPs were validated before and after imputation, respectively.

### Homozygous and heterozygous SNPs

Out of 62 million possible genotypes (462 samples × 134,528 sites), the average frequency of heterozygous SNPs was 25.32% (±5.6%) before the imputation and after the imputation, it increased to 27.70% (±5.2%). The average heterozygosity observed per chickens before imputation ranged from 8.30–44.69% and after the imputation between 11.38–44.67%. The proportion of heterozygous SNPs remained virtually unchanged before and after imputation among the lines/generations (Table 3).

### Principal component analyses

**Figure 1:**
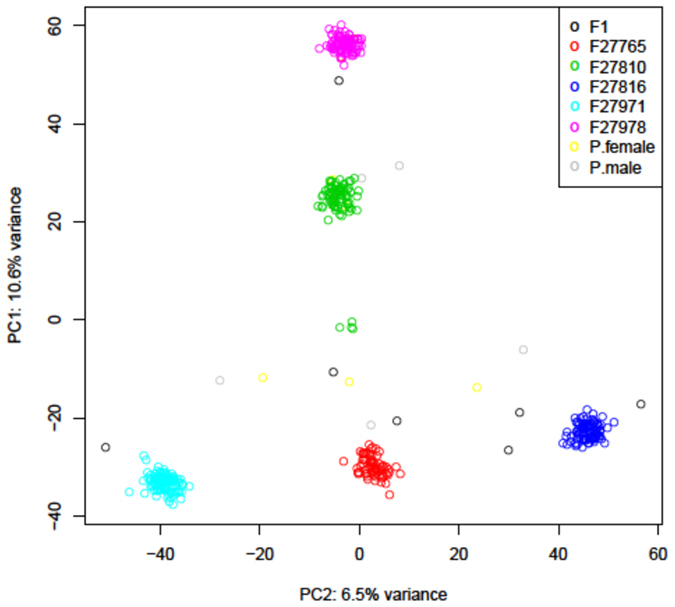
The 444 chickens from five F_2_ families and18 chickens from the parental lines (P. female and P. male - N = 10) and F_1_ generation (N = 8) shown in the 2D plane spanned by their first two principal components.

From the list of imputed genotypes we have conducted a principal component analysis (PCA), based on covariance, using Tassel v.5.2.26^39^ to check the F_2_ population structure. This plot was useful for visualizing internal structure explained by the variance from *PstI*-derived SNPs dataset of 134,528 SNPs using eigenvector-based multivariate analyses. Each individual lies in its proper group consistent with our F_2_ population structure composed by five F_2_ dame-based families (Fig. 1).

### Descriptive Statistics of Heritability

The genetic and residual variance for each trait and their genomic heritability are shown in Table 4. Heritabilities ranging from moderate to high, as is expected^40^,^41^, were observed for feed intake and body weights traits, respectively. Low heritabilities were observed for the traits evaluated in short period (between 35 and 41 days) as feed conversion, and feed efficiency, because they are complex traits influenced by several environmental factors^42^.

### Genome-wide association study

**Figure 2:**
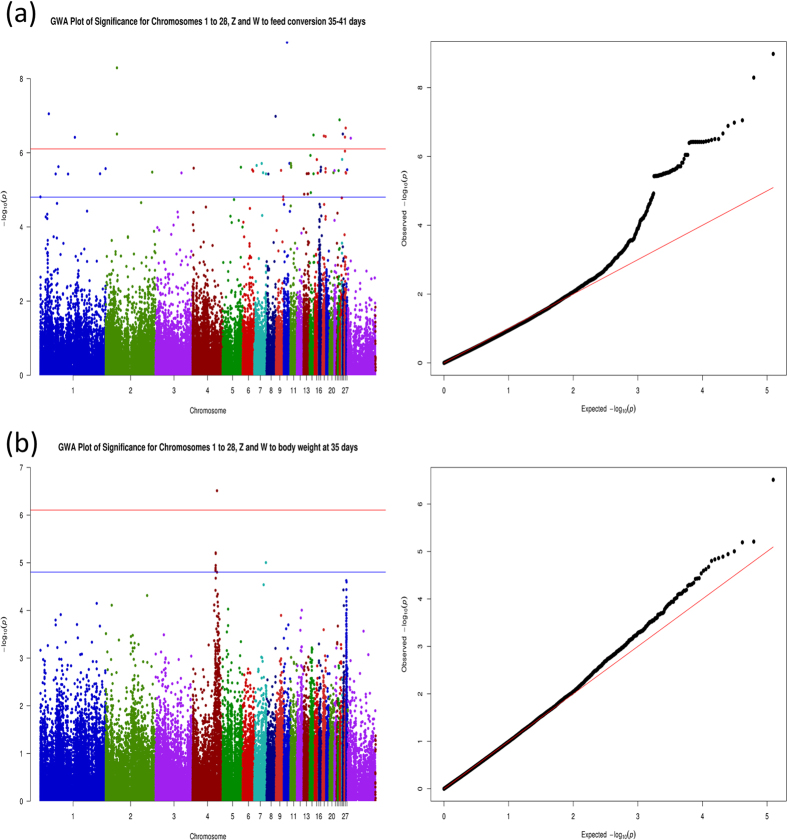
SNPs associated with feed conversion adj35. (**a**) and body weight at 35 days (**b**) are presented by Manhattan (left side) and QQ (right side) plots. The y-axis is shown as -log10 (p-value). On the left, the red line indicates genome-wise association (P < 7.86E-07) and the blue line, suggestive genome-wise association (P < 1.57E-05) with the respective trait. On the right side, the QQ-plots show the relation of normal theoretical quantiles of the probability distributions between expected (x-axis) and observed (y-axis) p-values from each respective associated trait (feed conversion adj35 and body weight at 35 days).

Twenty significant SNPs (P < 7.86E-07) were associated with feed conversion adjusted to body weight at 35 days (adj35) and one significant SNP associated with body weight at 35 days of age (Fig. 2). In addition to that, 92 suggestive (P < 1.57E-05) SNPs were associated with feed conversion adj35, feed intake adj35, feed efficiency adj35, birth weight, and body weight at 35 and 41 days of age (see Supplementary Spreadsheet S1 for the effects of associated SNPs; Manhattan and QQ plots are available on Supplementary Fig. S1).

### Linkage disequilibrium analysis

**Figure 3:**
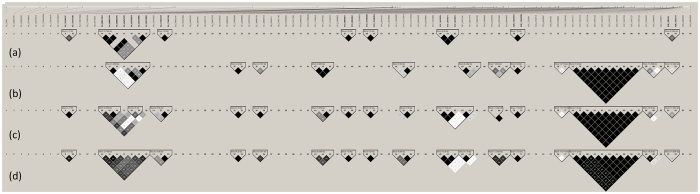
Haplotype blocks obtained by the solid spine of LD and family structure using Haploview 4.2. The header represents the physical position of each 94 SNPs selected on chicken genome (*Gallus gallus 4.0,* NCBI). The family structure is disposed as maternal CC (**a**) and paternal TT (**b**) parental pure lines, F_1_ (**c**) and F_2_ (**d**) generations. The black filled squares indicate r^2^ = 1 and square numbers represents r^2^*100 with black color gradient accordingly.

Seventeen haplotype blocks were generated from the associated and suggestively associated SNPs from the F_2_ population (see Fig. 3, Supplementary Fig. S2 for haplotypes details and Supplementary Table S1 to SNPs’ Mendelian descriptions). We noticed a standard block pattern between the SNPs that matched with the F_2_ population structure (Fig. 3). Interestingly, we have checked the genotype frequency of blocks formed by LD analysis to determine if the blocks were fixed or not in the parental lines. From the haplotype blocks, we checked the origin of the variation (fixed or variable) and frequency from F_2_ generation in the parental lines (Supplementary Table S2 and Supplementary Fig. S2 for a more detailed description of frequencies). We also determined the advantageous haplotype for each trait in the F_2_ generation (Table 5). This information enabled us to identify from which parental line (TT or CC) comes the genotypic variation observed in F_2_ for each block. All blocks with r^2^ > 0.56 had the most frequent haplotype agreeing with the advantageous phenotype in the F_2_ individuals (Fig. 3; Supplementary Fig. S2 and Supplementary Table S2), and this advantageous haplotype (lower feed conversion and higher values of other evaluated traits) was fixed in one of the parental lines, except in blocks 2 and 13. This information is also available for each genome-wide suggestive and/or associated SNPs in Supplementary Spreadsheet S1, as well as the number of genotype observations obtained per SNP.

### QTL overlapping SNPs

**Figure 4:**
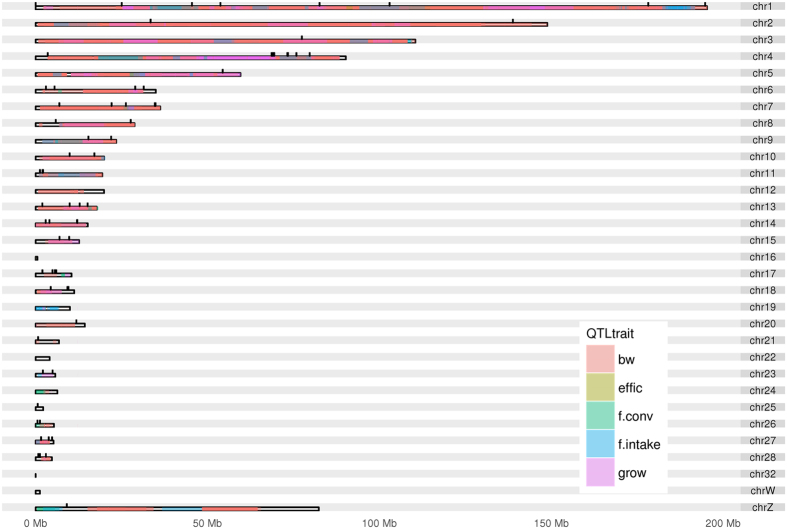
Karyotype of the QTLs (from Animal QTLdb) distribution regions of the chicken genome overlapping suggestive and significant SNPs associated with performance traits (black marks). These QTLs were mapped for body weight (bw), postnatal growth (grow), feed intake (f.intake), feed conversion (f.conv), and feed efficiency (effic). The colors becoming denser according to the number of QTL previously mapped superposing each other.

Through Animal QTLdb, we have selected all the 1,458 known QTLs^43^ mapped for body weight, feed efficiency, feed conversion and growth, all evaluated in different chicken lines and ages. Out of those, we have observed that 253 QTLs overlapped with 81 of the 94 associated and suggestively associated SNPs with performance traits obtained from the GWAS in this study: 206 QTLs associated with body weight, 39 with postnatal growth, 4 with feed intake, 3 with feed conversion, and 1 with feed efficiency. The complete QTL list that overlapped with these regions can be seen in the Supplementary Table S3 and the graphical representation of the suggestive and significant SNPs distribution in relation to the QTLs can be observed in Fig. 4.

## Discussion

For better understanding of complex traits control in a segregating F_2_ population, our research group have focused the attention on genetic association and linkage analyses using different approaches, as: candidate genes^14^,^15^,^16^,^17^ and QTL mapping^3^,^4^,^5^,^6^,^7^, respectively, and more recently, NGS approaches^27^,^28^. We have presented here the first study using a higher density of SNPs in this F_2_ population with GWAS purpose. Therefore, we have optimized a method called CornellGBS in chickens^35^ to overcome the concept of pre-designed panels, since we planned a method for genotyping efficiently a specific dataset of SNPs in our specific population.

CornellGBS is a widely employed method to genotype large genomes of model and non-model species exploring important regions in the genome^34^ as microchromosomes^36^,^37^, as previously mentioned. This is due to the high coverage of *tags* (contigs) depending on the number of sequenced individuals of the reduced genome by restriction enzyme cleavage providing a specific SNP profile^35^. The CornellGBS technique was previously developed for inbreeding population and it is known by its general low sequencing coverage, which can cause significant loss of SNPs, mainly heterozygous^39^. For our outbreed population, we used a reasonable multiplex of individuals (~48 animals per lane of Illumina flowcell) to maintain a reasonable sequencing coverage per individual (~5X). We also reduced the loci call rate and use imputation to increase the number of SNPs genotyped. The reduction in the loci call rate was also applied in a recent study that used the same *PstI* restriction enzyme to cleave the cattle genome^44^. Furthermore, it was already mentioned that the combination of GBS and imputation of missing internal SNPs in haplotype blocks procedures can promote a cost reduction by allowing further reduction of the filtering criteria or sequencing coverage without causing losses in SNP calls^34^. Using this strategy, we doubled the number of SNPs, successfully imputing all lost genotypes (increasing the individual call rate to 100%), the validation ratio remained > 90%, and the percentage of heterozygous genotypes in our population had an increase of approximately 2% after the imputation.

The use of the GBS SNP panel for GWAS in our outbred F_2_ crosses resulted in 20 SNPs associated (P < 7.86E-07) with feed conversion adj.35, one SNP associated with body weight at 35 days (BW35) and other 93 SNPs suggestively associated (P < 1.57E-05) with different performance traits (Table 1). Additionally, we noticed that all the evaluated traits, presented an up deviation of the theoretical quantiles (Fig. 2 and Supplementary Fig. S1) of the probability distributions between expected and observed p-values, indicating the existence of QTLs. These results corroborated the Manhattan plot peaks of associated SNPs, indicating that these traits had part of the phenotypic variation significantly explained by the genetic component^45^. Interestingly, we detected association for several new QTLs located in microchromosomes (GGA11-28). This was only possible because of the distribution of the SNPs used. From our set of SNPs, 38.93% are located in large chromosomes (GGA1-5), 14.15% in intermediate size (GGA6-10), and most, 46.90% are located in microchromosomes (GGA11-28), confirming the microchromossome enrichment mentioned before^35^. Feed conversion, for exemple, had a high number of significant SNPs (P < 7.86E-07), mainly located in microchromosomes (GGA8, 10, 14, 18, 23, 26, and 27). However, for this trait, the SNP peaks observed by Manhatan plot, in large and intemediate size chromossomes (Fig. 2a), were not well defined, as is usually observed for QTLs peaks^46^. We believe that this is explained by the SNP profile used in this study, which has a lower density of SNPs on large chromosomes compared to microchromosomes^35^. Moreover, feed intake is a complex trait subject to a high residual effect and controlled by several genes with a small effect, which require a large sample size to detect associations^47^,^48^. This small effect also was previously attributed to the short period used to measure this trait (between 35 and 41 days of age) impairing the animal adaptation to the new enviromental condiction^4^. On this account, reliable QTLs for this trait were detected in studies that used a larger sampling size (1,534 individuals) for a longer feed intake evaluation period (~4 weeks)^49^,^50^ than used in this study.

The GBS strategy can result in clusters of SNPs next to each other^35^. In order to better define the QTL regions we performed LD analysis. The block pattern between the SNPs matched with our F_2_ population structure^51^ (Fig. 3). This allowed us to define possibilities for genetic selection of the lines that did not present the genotype fixed giving attention to the different phenotypic abilities between the CC layer line and the TT broiler line^5^,^16^,^51^. As for example the blocks 2 and 13 (consider Fig. 3(d) for block numbers), which had variable genotypes for both the CC and the TT lines, and the paternal line presented the favorable genotype most frequent in both cases (see Supplementary Fig. S2 and Fig. 3).

Also is important to check if these SNPs are within QTL regions previously published. In the past years, many studies identified QTLs associated with performance traits in different chickens populations^3^,^4^,^5^,^6^,^8^,^9^,^10^,^11^,^12^,^13^,^17^,^52^,^53^ (1,458 QTLs described in the Animal QTLdb) aiming to map loci that control these traits. Recently, to better understand these loci, studies have also applied GWAS with performance traits in chickens^13^,^26^,^54^. The validation of single SNP position obtained by GWAS overlapping with QTL regions can confirm interesting genomic regions to explore. From the 94 genome-wise associated and suggestively associated SNPs with the performance traits analyzed in this study, most of them were fairly distributed in mapped QTL regions in the chicken genome (Fig. 4). Only 13 SNPs did not overlap with QTL regions previously mapped. From these 13 SNPs, one was located on chromosome 1, one in chromosome 8 (GGA1 and 8), one in the Z sex chromosome (GGAZ) and 10 were located in microchromosomes (GGA17, 18, 20, 25, 27 and 28), which confirms the microchromosome enrichment profile obtained by this approach^35^ and suggests novel QTLs to be explored in these regions. It is also important to mention that most of these 13 SNPs (those located on GGA1, 8, 18, 20, 25 and 27) were associated (P < 7.86E-07) or suggestively associated (P < 1.57E-05) with feed conversion adj35, one with feed efficiency adj35 (GGA17), and two with body weight at 41 days (GGA27) (see Supplementary Spreadsheet S1 for details). The genes where these SNPs are located are mainly related with cell cycle and metabolic pathways (according to the Reactome pathways - http://www.reactome.org/PathwayBrowser) and were within introns, upstream and downstream of these genes (see Supplementary Table S4 for functional annotation).

Despite the importance of the overlap test performed here, previous studies in QTL mapping usually had large confidence intervals (>1 Mbps) and often encompassing several genes, making difficult the selection of candidate genes^55^. Therefore, we also checked the overlap of these SNPs only with QTLs mapped using specifically the same traits and the same F_2_ population^4^,^5^,^14^,^17^ used in this study. From 23 different QTL intervals, we identified 12 SNPs overlapping with seven of them (see the QTLs bolded in Supplementary Tables S3 and S5 to check the QTL list). It is worth mentioning the SNPs located near the QTL regions, or flanking regions (see Supplementary Fig. S3). On GGA1, for exemple one SNP (marker 6, see Supplementary Spreadsheet S1) associated with feed intake (P = 3,83E-07) overlaped with one QTL mapped for the same trait^50^ in another population, and also with 6 QTLs mapped for body weight at different ages^56^ and one with feed efficiency^57^. On the other hand, on GGA4, a well studied chromosome in chickens^4^,^9^,^11^,^12^,^14^,^16^,^20^,^23^,^24^,^25^,^54^,^58^,^59^,^60^,^61^,^62^,^63^, we identified three SNPs composing the haplotype 3 (markers 21–23), in which one was associated with BW35 (P < 7.86E-07) and suggestively associated with BW41 (P < 1.57E-05), and two SNPs suggestively associated with BW41 (P < 1.57E-05). These three SNPs overlapped with one QTL region previously mapped in this same population for these same traits^4^ (QTL_IDs from ChickenQTLdb = 7157; 7162 and 7185). The boundary SNPs from haplotypes 2 and 3 (Fig. 3(d)) are separated by a short distance (less than 4 Mbps), but these QTLs are not linked, beside they have effect on the same traits (BW35 and BW41) (see Supplementary Spreadsheet S1) in our F_2_ population. It is worth to mention, the haplotype 2 that overllaped with QTLs mapped for different BW in different ages^52^,^53^,^64^,^65^,^66^,^67^ and growth^13^,^52^,^67^ traits in different populations.

To the best of our knowledge, we showed the application of the CornellGBS *PstI*-derived SNPs to a GWAS for the first time in chickens. We showed a strategy, changing filtering criteria and subsequent genotype imputation, to increase the number of reliable SNPs to be analyzed. We found 13 SNPs indicating new regions associated with performance traits, mainly in microchromosomes, that have not been previously reported. We improved the available information about loci controlling performance traits and we refined these regions to discover novel candidate regions to be explored. Finally, by demonstrating that GBS is a valid strategy for QTL mapping in a species that has genome sequence and SNP panel available, we can argue the validity of GBS in species without genome resources.

## Methods

All experimental protocols employed in the present study that relate to animal experimentation were performed in accordance with the resolution number 010/2012 approved by the Embrapa Swine and Poultry Ethics Committee on Animal Utilization to ensure compliance with international guidelines for animal welfare.

### Animals and Phenotypes

This study was conducted using 464 chickens from a F_2_ populations originated from a reciprocal cross-experimental population from Embrapa Swine and Poultry National Research Center, Concórdia, SC, Brazil. We also included in the analysis, 10 chickens from their parental lines TT and CC (5 from each one), and eigth from the F_1_ generation. This F_2_ population was developed for QTL mapping studies, and was originated from the crossing of seven males from a broiler line (TT) and seven females from a layer line (CC), resulting in seven full-sib families (F_1_ generation). Then, twenty-one F_1_ females were artificially inseminated with seven F_1_ males (3:1 ratio) to generate the F_2_. The F_2_ population comprised seven paternal half-sib families composed of three full-sib families of approximately 100 individuals each, produced in 17 hatches, totaling 2,063 F_2_ chickens^51^. For this study, we selected the five most informative families based on the previously QTL studies^4^,^16^,^63^.

The TT broiler line was selected over six generation to improve body weight, feed conversion, carcass and breast yields, viability, fertility, eclodibility, reduction of abdominal fat and metabolic syndromes^51^. The CC layer line was selected over eight generation to improve egg production, egg weight, feed conversion, viability, sexual maturity, fertility, eclodibility, egg quality and reduction of body weight^51^.

The F_2_ chickens were reared as broilers with free access to corn and soybean meal-based diet and water up to 42 days of age. From 35 to 41 days, they were transferred to cages to collect feed intake, and to compute the conversion and efficiency. Body weight was recorded at 1 (birth weight), 35, 41 and 42 (after fasting) days of age. The body weight (BW) at 41 days of age was collected at the end of the conversion measurement and, BW42 days was collected after 6-h fasting period and transportation to the slaughterhouse. More details were previously provided^5^,^16^,^51^. We analysed these six performance traits in this study (feed conversion, feed intake and feed efficiency between 35 to 41 days, birth weight, and body weight at 35 and 41 days of age). A total of 23 missed values from the selected traits were imputed by mean using Tassel v.5.2.26 tool^39^ among the 446 F_2_ animals selectect to be evaluated in this study.

### DNA, genotypic data and imputation

Genomic DNA was cleaved with the *PstI* enzyme, ligated to adapters with barcodes identifying individual animals, and then sequenced on Illumina platform. After filtering parameters were applied, 134,528 SNPs were identified from 462 individuals in our experimental population of chickens using minimum minor allele frequency (mnMAF) of 1%; minimum taxon coverage (mntCov) of 20% and minimum site coverage (mnScov) of 70% filter parameters. All procedures to obtain the data were previously described^35^. After filtering parameters, the number of missing genotypes increased from 0.9 to 5.8 million. This number represents SNPs identified multiplied for the number of individuals genotyped (67,096 derived *PstI*-SNPs × 462 individuals using 90% of loci call rate and 134,528 derived *PstI*-SNPs x 462 individuals using 70% of loci call rate). The imputation of missing genotypes was performed using Beagle 4.1 software^38^ using default parameters, which uses empirical LD model. This model adapts to the local structure in the data using iterative approach to haplotype phasing in which an initial prediction of haplotype phase is made, then the model is fit, and improved estimates of haplotype phase are obtained and the model is refit^38^.

### SNPs validation

The validation was performed by the comparison of the filtered 134,528 *PstI*-derived SNPs dataset with the 600 K Affymetrix® HD genotyping array SNP dataset using five individuals from F_2_-7810 family. The SNPs in both datasets were located on GGA1-28, 32, W and Z chromosomes on the Gallus-gallus-4.0 reference genome. The validation standards used in this study were based on a methodology previously proposed^68^ considering chromosomal positions and genotype concordances. With these concordances, we estimate the accuracy for homozygous and heterozygous SNPs.

### Principal component analysis

The principal component analyses (PCA) of imputed genotype data were performed using Tassel v.5.2.26 39 considering five principal components. This tool transforms a set of correlated variables into successive orthogonal PCs accounted for the maximum variance providing a way to highlight groups of individuals differing at the level of minor allele frequency^39^. The PCA graph was produced using the plot function in R 2.13.2 software (http://www.r-project.org/).

### Genome-Wide Association Study

The compressed mixed linear model (MLM) implemented in TASSEL v.5.2.26 software^45^ was used for GWAS. The following form represents the statistical model:


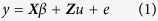


where y is the vector of the dependent variables, *β* is the vector containing fixed effects, including the sex (male/female), hatch (1–17) and SNP (−1, 0, 1) effects, and the covariate body weight at 35 days for traits measured from 35 to 41 days of age (feed intake, feed efficiency and feed conversion); *u* is the vector of random additive genetic effects from background QTL for individuals, *X* and Z are design matrices, and *e* is the vector of random residuals. We assumed that *u* and *e* vectors are normally distributed with null mean and variance of


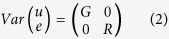


where 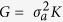; 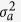 is an unknown additive genetic variance and *K* is the kinship (co-ancestry) matrix calculated from SNPs and provided by the same software using centered identity by state (IBS) method. For the residual effect, homogeneous variance was assumed, with 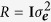, where **I** is an identity matrix and 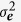
is the unknown residual variance. The Restricted Maximum Likelihood (REML) estimates of 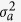
and 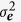
were obtained by the Efficient Mixed-Model Association (EMMA) algorithm^69^. Heritability (h^2^) was calculated as the ratio of the additive genetic variance (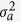) to the phenotypic variance (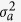
+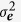). Tassel program does not provide the standard errors of the estimates. Thus, standard errors were estimated using the REML method with an average information (AI) algorithm by AIREMLF90 software^70^. Standard errors for additive genetic and residual variance were computed as square roots of diagonal elements of the inverse of the average information matrix. For Heritability, standard deviations obtained from the repeated sampling approach were considered as their standard error^71^. Each SNP allele was fit as a separate class with heterozygotes fit as additional SNP classes. The total SNP effect was not decomposed in additive and dominance effects but tested for overall significance^45^

Quantile-quantile (Q-Q) plots for each trait and Manhattan plots of genome-wide association analyses were performed in R using ggd.qqplot and Manhattan functions. The thresholds were corrected for multiple testing (Bonferroni) established by the estimated number of independent SNPs and LD blocks (pairwise r^2^ values > 0.40)^72^ that was 63,640 SNPs. We set two thresholds from our data: P < 1.57E-05 (1/63,640) for suggestive significance and P < 7.86E-07 (0.05/63,640) for 5% genome-wise significance level^54^,^60^.

### Linkage disequilibrium analysis

The linkage disequilibrium (LD) analysis was performed with Haploview 4.2^73^ as well as the LD graphs. To perform the pairwise comparison of ours SNPs considering 1 Mb apart, we selected 94 genome-wise associated and suggestively associated SNPs with the traits analyzed in this study (feed conversion, feed intake, feed efficiency, and weight gain between 35–41 days of age, birth weight and body weight at 35 and 41 days). We have defined the haplotype blocks by the solid spine of LD and the family structure of parental lines (CC and TT pure lines), F_1_ and F_2_ generations (Fig. 3) using Haploview 4.2.

### QTL overlapping with SNPs

The QTL data was obtained from QTLdb^43^ and the overlapping test was performed using the GenomicRanges package from R 3.3.1 software (http://www.r-project.org/). For the data presentation, we designed a figure using ggplot2 package karyogram layout from R 3.3.1 software.

## Additional Information

**How to cite this article**: Pértille, F. *et al*. Genome-wide association study for performance traits in chickens using genotype by sequencing approach. *Sci. Rep.* **7**, 41748; doi: 10.1038/srep41748 (2017).

**Publisher's note:** Springer Nature remains neutral with regard to jurisdictional claims in published maps and institutional affiliations.

## Supplementary Material

Supplementary Information

Supplementary Spreadsheet S1

## Figures and Tables

**Table 1:** Means, standard deviations (SD), maximum (max) and minimum (min) values for performance traits of 444 individuals from the F_2_ population.

Traits	Average (SD)	Max	Min
Feed conversion from 35-41 days	2.87±0.71	7.55	1.52
Feed intake from 35–41 days (g)	603.43±139.74	1176.00	258.00
Feed efficiency from 35–41 days	0.36±0.07	0.66	0.13
Birth weight (g)	44.96±4.15	55.50	34.60
Body weight at 35 days (g)	789.86±138.60	1309.00	480.00
Body weight at 41 days (g)	1006.82±188.34	1686.00	407.00

^*^g is the weight measured in grams.

**Table 2:** Assessment of genotype concordance between 134,528 *PstI*-derived filtered SNPs before and after imputation and genotyped SNPs dataset from 600 K Affymetrix® HD genotyping array from five F_2_ individuals (F_2_-7810 family); and genotype validation percentages for homozygous and heterozygous SNPs.

F_2_-7810 family	SNP type	Before Imputation		After Imputation		Total validated genotypes*	
		Concordant physical position^†^	Validated genotypes* (%)	Concordant physical position^†^	Validated genotypes *(%)	Before Imputation *(%)	After Imputation *(%)
ID-1209	homoz^1^	2,410	97.67	2,493	97.47	93.08	92.93
	heteroz^2^	1,323	85.56	1,370	85.47		
ID-1786	homoz^1^	2,379	97.73	2,427	97.65	94.04	93.96
	heteroz^2^	1,422	88.60	1,440	88.47		
ID-1787	homoz^1^	2,296	97.99	2,356	97.87	93.72	93.42
	heteroz^2^	1,470	87.68	1,512	87.17		
ID-2301	homoz^1^	1,589	98.48	2,409	97.50	87.62	87.67
	heteroz^2^	948	70.25	1,457	72.06		
ID-786	homoz^1^	2,383	97.98	2,438	97.74	90.57	90.33
	heteroz^2^	1,395	78.63	1,422	78.34		

^1^Homozygous genotype; ^2^heterozygous genotype; ^†^number of concordant physical positions between both datasets used for validation (from CornellGBS vs 600 K Affymetrix® HD genotyping array); *is the percentage of † that had concordant genotypes.

**Table 3:** SNP heterozygosity of genotyped populations (parental, F_1_ and F_2_ generations) after and before imputation.

Population	Number of individuals	Proportion of heterozygous (SD)	
		Before Imputation	After Imputation
Parental CC	5	0.16 ± 0.01	0.17 ± 0.01
Parental TT	5	0.21 ± 0.01	0.22 ± 0.01
F1	8	0.28 ± 0.09	0.29 ± 0.08
F2-7765	72	0.29 ± 0.03	0.29 ± 0.03
F2-7810	82	0.28 ± 0.04	0.28 ± 0.04
F2-7816	94	0.30 ± 0.08	0.30 ± 0.08
F2-7971	100	0.25 ± 0.05	0.26 ± 0.04
F2-7978	96	0.26 ± 0.04	0.27 ± 0.03

**Table 4:** Genetic and residual variances, and genomic heritability for each trait analyzed in this study.

Traits	Var_genetic ± SE	Var_error ± SE	Heritability ± SE
Feed conversion 35adj	0.0045 ± 0.00282	0.4363 ± 0.01797	0.01 ± 0.006
Feed intake from 35adj	1492.3 ± 855.94	7296.0 ± 830.22	0.17 ± 0.094
Feed efficiency 35adj	0.0005 ± 0.00002	0.0036 ± 0.00002	0.11 ± 0.005
Birth weight	2.5719 ± 0.50599	3.1009 ± 0.33925	0.45 ± 0.073
Body weight at 35 days	10403 ± 1575.9	1879.9 ± 785.7	0.85 ± 0.073
Body weight at 41 days	16790 ± 2936.7	5639 ± 1701.1	0.75 ± 0.087

35adj is adjusted to body weight at 35 days, SE is the standard error, and for Heritability, the SE is the standard deviations obtained from a repeated sampling approach (see M&M section for detailed information).

**Table 5:** TagSNP significance levels from MLM analyses of each 17 blocks obtained by solid spine of LD in the F_2_ population using Haploview 4.2.

HaplotypeN° (GGA)	Haplotype	Haplotype Frequency (%)				Trait	p-value	add p-value	dom p-value
		Parental CC	Parental TT	F_1_	F_2_				
1(2)	**AC^†^**	50	100	75	84	eff.adj35	1.36E-05*	2.46E-03*	2.11E-05*
						f.conv.adj35	5.11E-09**	2.04E-07**	1.05E-09**
2(4)	**CAGAATG†**	0	60	31	42	bw35	6.19E-06*	3.26E-05*	1.13E-02*
	AGTGTCA	80	0	0	40	bw41	4.70E-06*	5.31E-06*	1.25E-02*
3(4)	**TAG^†^**	0	80	31	44	bw35	3.08E-07**	1.95E-07**	8.31E-02*
	**CGA**	90	0	0	38	bw41	1.40E-06*	1.16E-06*	6.62E-02*
4(7)	**GC†**	100	70	94	94	f.conv.adj35	1.94E-06*	3.38E-07**	7.30E-07**
5(7)	AG	100	80	88	93	bw35	9.91E-06*	3.63E-06*	4.53E-03*
						f.conv.adj35	3.70E-06*	6.96E-07**	1.18E-06*
6(11)	**GGA†**	100	90	88	93	f.conv.adj35	1.94E-06*	3.98E-07**	7.29E-06*
7(13)	**CC†**	80	90	75	78	f.conv.adj35	3.67E-06*	5.76E-07**	9.02E-07*
8(13)	**TC^†^**	90	100	88	90	f.conv.adj35	3.63E-06*	7.39E-07**	7.51E-07**
9(14)	**TGC^†^**	100	60	68	85	f.conv.adj35	3.31E-07**	8.88E-08**	6.11E-08**
10(17)	**CGT^†^**	70	100	88	85	f.conv.adj35	3.15E-06*	5.18E-07**	1.48E-06*
11(17)	**CC^†^**	100	90	94	95	f.conv.adj35	2.94E-06*	7.38E-07**	6.73E-07**
12(18)	**ACC^†^**	90	70	94	93	f.conv.adj35	3.31E-06*	5.16E-07**	3.09E-06*
13(20)	**TC^†^**	70	90	75	78	f.conv.adj35	3.45E-06*	5.45E-07**	1.07E-06*
14(26)	**CA^†^**	100	60	75	79	f.conv.adj35	3.12E-07**	4.54E-08**	1.75E-06*
						f.int.adj35	1.04E-05*	1.36E-04*	1.73E-06*
15(27)	**CAGGGAACCA^†^**	100	90	88	95	f.conv.adj35	9.00E-07*	3.26E-07**	1.77E-05*
16(27)	**CG^†^**	100	80	63	94	f.conv.adj35	2.15E-07**	1.38E-07**	4.91E-08**
17(28)	CA^†^	70	10	44	35	bw41	2.22E-06*	4.57E-07**	1.58E-01*
	TG	20	50	31	30				
	**CG**	10	40	25	34				

(GGA) is the *Gallus gallus* chromosome number; ^†^Most frequent haplotype in the population; bolded is the advantage haplotype to individuals compared to the assessed trait; additive (add) and dominant (dom) p-values; *suggestive genome-wise significance (P < 1.57E-05) and **genome-wise significance (P < 7.86E-07). Abbreviations: eff.adj35 (feed efficiency adjusted for body weight at 35 days), f.conv.adj35 (feed conversion adjusted for body weight at 35 days), f.int.adj35 (feed intake adjusted for body weight at 35 days), all between 35 and 41 days of age and adjusted for 35 days; bw35, bw41, bw42 is body weight at 35, 41 and 42 days of age, respectively.

## References

[b1] Fulton J. E. Genomic selection for poultry breeding. Anim. Front. 2, 30–36 (2012).

[b2] Blackburn H. D. The National Animal Germplasm Program: challenges and opportunities for poultry genetic resources. Poult. Sci. 85, 210–215 (2006). 16523615 10.1093/ps/85.2.210

[b3] Ambo M. . Genetic linkage maps of chicken chromosomes 6, 7, 8, 11 and 13 from a Brazilian resource population. Sci. Agric. 65, 447–452 (2008).

[b4] Ambo M. . Quantitative trait loci for performance traits in a broiler x layer cross. Anim. Genet. 40, 200–8 (2009). 19170675 10.1111/j.1365-2052.2008.01824.x

[b5] Nones K. . Mapping QTLs on chicken chromosome 1 for performance and carcass traits in a broiler x layer cross. Anim. Genet. 37, 95–100 (2006). 16573522 10.1111/j.1365-2052.2005.01387.x

[b6] Nones K. . Quantitative trait loci associated with chemical composition of the chicken carcass. Anim. Genet. 43, 570–6 (2012). 22497237 10.1111/j.1365-2052.2012.02321.x

[b7] Campos R. L. R. . Quantitative trait loci associated with fatness in a broiler-layer cross. Anim. Genet. 40, 729–36 (2009). 19466938 10.1111/j.1365-2052.2009.01910.x

[b8] Tatsuda K. & Fujinaka K. Genetic mapping of the QTL affecting body weight in chickens using a F 2 family. Br. Poult. Sci. 42, 333–337 (2001). 11469552 10.1080/00071660120055296

[b9] Koning D. J. de . Quantitative trait locus detection in commercial broiler lines using candidate regions. J Anim Sci 81, 1158–1165 (2003). 12772842 10.2527/2003.8151158x

[b10] Ikeobi C. O. . Quantitative trait loci for meat yield and muscle distribution in a broiler layer cross. Livest. Prod. Sci. 87, 143–151 (2004).

[b11] Nassar M. K., Goraga Z. S. & Brockmann G. a. Quantitative trait loci segregating in crosses between New Hampshire and White Leghorn chicken lines: II. Muscle weight and carcass composition. Anim. Genet. 43, 739–45 (2012). 22497436 10.1111/j.1365-2052.2012.02344.x

[b12] Nassar M. K., Goraga Z. S. & Brockmann G. a. Quantitative trait loci segregating in crosses between New Hampshire and White Leghorn chicken lines: III. Fat deposition and intramuscular fat content. Anim. Genet. 44, 62–8 (2013). 22607452 10.1111/j.1365-2052.2012.02365.x

[b13] Zhou H., Deeb N., Evock-Clover C. M., Ashwell C. M. & Lamont S. J. Genome-wide linkage analysis to identify chromosomal regions affecting phenotypic traits in the chicken. I. Growth and average daily gain. Poult. Sci. 85, 1700–11 (2006). 17012159 10.1093/ps/85.10.1700

[b14] Felício A. M. . Polymorphisms in FGFBP1 and FGFBP2 genes associated with carcass and meat quality traits in chickens. Genet. Mol. Res. 12, 208–22 (2013). 23408407 10.4238/2013.January.24.13

[b15] Felício a. M. . Identification and association of polymorphisms in CAPN1 and CAPN3 candidate genes related to performance and meat quality traits in chickens. Genet. Mol. Res. 12, 472–82 (2013). 23420372 10.4238/2013.February.8.12

[b16] Pértille F. . Identification of polymorphisms associated with production traits on chicken (Gallus gallus) chromosome 4. Genet. Mol. Res. 14, 10717–10728 (2015). 26400301 10.4238/2015.September.9.11

[b17] Boschiero C. . Association of IGF1 and KDM5A polymorphisms with performance, fatness and carcass traits in chickens. J. Appl. Genet. 54, 103–12 (2013). 23275255 10.1007/s13353-012-0129-6

[b18] Shen X. . The GTPase activating Rap/RanGAP domain-like 1 gene is associated with chicken reproductive traits. PLoS One 7, e33851 (2012). 22496769 10.1371/journal.pone.0033851 PMC3322132

[b19] Nie Q.-H., Zhang X.-Q. & Lei M.-M. Single nucleotide polymorphism and its use in chicken QTL mapping. Yi Chuan 25, 729–34 (2003). 15639971

[b20] Sun Y. . The identification of 14 new genes for meat quality traits in chicken using a genome-wide association study. BMC Genomics 14, 458 (2013). 23834466 10.1186/1471-2164-14-458 PMC3707761

[b21] Morris G. P. . Population genomic and genome-wide association studies of agroclimatic traits in sorghum. Proc. Natl. Acad. Sci. USA 110, 453–8 (2013). 23267105 10.1073/pnas.1215985110 PMC3545811

[b22] Park M. N. . Genome-wide Association Study of Chicken Plumage Pigmentation. Asian-Australasian J. Anim. Sci. 26, 1523–8 (2013). 10.5713/ajas.2013.13413 PMC4093824 25049737

[b23] Luo C. . Genome-wide association study of antibody response to Newcastle disease virus in chicken. BMC Genet. 14, 42 (2013). 23663563 10.1186/1471-2156-14-42 PMC3654938

[b24] Luo C. . Genetic parameters and genome-wide association study of hyperpigmentation of the visceral peritoneum in chickens. BMC Genomics 14, 334 (2013). 23679099 10.1186/1471-2164-14-334 PMC3663821

[b25] Sun Y. . Genome-wide linkage analysis and association study identifies loci for polydactyly in chickens. G3 (Bethesda). 4, 1167–72 (2014). 24752238 10.1534/g3.114.011338 PMC4065260

[b26] Xie L. . Genome-wide association study identified a narrow chromosome 1 region associated with chicken growth traits. PLoS One 7, e30910 (2012). 22359555 10.1371/journal.pone.0030910 PMC3281030

[b27] Moreira G. C. M. . Variant discovery in a QTL region on chromosome 3 associated with fatness in chickens. Anim. Genet. 46, 141–147 (2015). 25643900 10.1111/age.12263

[b28] Godoy T. F. . SNP and INDEL detection in a QTL region on chicken chromosome 2 associated with muscle deposition. Anim. Genet. 46, 158–163 (2015). 25690762 10.1111/age.12271

[b29] Ahsan M. . Identification of candidate genes and mutations in QTL regions for chicken growth using bioinformatic analysis of NGS and SNP-chip data. Front. Genet. 4, 1–8 (2013). 10.3389/fgene.2013.00226 PMC3817360 24204379

[b30] Roux P.-F. . Re-Sequencing Data for Refining Candidate Genes and Polymorphisms in QTL Regions Affecting Adiposity in Chicken. PLoS One 9, e111299 (2014). 25333370 10.1371/journal.pone.0111299 PMC4205046

[b31] Li X. . Using targeted re-sequencing for identification of candidate genes and SNPs for a QTL affecting the pH value of chicken muscle. bioRxiv 5, 17186 (2015). 10.1534/g3.115.020552 PMC4592991 26276381

[b32] Groenen M. a. M. . The development and characterization of a 60K SNP chip for chicken. BMC Genomics 12, 274 (2011). 21627800 10.1186/1471-2164-12-274 PMC3117858

[b33] Kranis A. . Development of a high density 600K SNP genotyping array for chicken. BMC Genomics 14, 59 (2013). 23356797 10.1186/1471-2164-14-59 PMC3598943

[b34] He J. . Genotyping-by-sequencing (GBS), an ultimate marker-assisted selection (MAS) tool to accelerate plant breeding. Front. Plant Sci. 5, 484 (2014). 25324846 10.3389/fpls.2014.00484 PMC4179701

[b35] Pértille F. . High-throughput and Cost-effective Chicken Genotyping Using Next-Generation Sequencing. Sci. Rep. 6, 26929 (2016). 27220827 10.1038/srep26929 PMC4879531

[b36] Habermann F. a. . Arrangements of macro- and microchromosomes in chicken cells. Chromosome Res. 9, 569–84 (2001). 11721954 10.1023/a:1012447318535

[b37] Smith J. . Differences in gene density on chicken macrochromosomes and microchromosomes. Anim. Genet. 31, 96–103 (2000). 10782207 10.1046/j.1365-2052.2000.00565.x

[b38] Browning S. R. & Browning B. L. Rapid and accurate haplotype phasing and missing-data inference for whole-genome association studies by use of localized haplotype clustering. Am. J. Hum. Genet. 81, 1084–97 (2007). 17924348 10.1086/521987 PMC2265661

[b39] Glaubitz J. C. . TASSEL-GBS: a high capacity genotyping by sequencing analysis pipeline. PLoS One 9, e90346 (2014). 24587335 10.1371/journal.pone.0090346 PMC3938676

[b40] Gaya L. G. . Heritability and Genetic Correlation Estimates for Performance and Carcass and Body Composition Traits in a Male Broiler Line. Poult. Sci. 85, 837–843 (2006). 16673760 10.1093/ps/85.5.837

[b41] Rovadoscki G. A. . Genetic parameters for growth characteristics of free-range chickens under univariate random regression models. Poult. Sci. 95, 1989–1998 (2016). 27208151 10.3382/ps/pew167

[b42] Reyer H., Hawken R., Murani E., Ponsuksili S. & Wimmers K. The genetics of feed conversion efficiency traits in a commercial broiler line. Sci. Rep. 5, 16387 (2015). 26552583 10.1038/srep16387 PMC4639841

[b43] Hu Z.-L., Park C. A., Wu X.-L. & Reecy J. M. Animal QTLdb: an improved database tool for livestock animal QTL/association data dissemination in the post-genome era. Nucleic Acids Res. 41, D871–D879 (2013). 23180796 10.1093/nar/gks1150 PMC3531174

[b44] De Donato M., Peters S. O., Mitchell S. E., Hussain T. & Imumorin I. G. Genotyping-by-sequencing (GBS): a novel, efficient and cost-effective genotyping method for cattle using next-generation sequencing. PLoS One 8, e62137 (2013). 23690931 10.1371/journal.pone.0062137 PMC3656875

[b45] Bradbury P. J. . TASSEL: Software for association mapping of complex traits in diverse samples. Bioinformatics 23, 2633–2635 (2007). 17586829 10.1093/bioinformatics/btm308

[b46] Fragomeni B. D. O. . Changes in variance explained by top SNP windows over generations for three traits in broiler chicken. Front. Genet. 5, 1–7 (2014). 25324857 10.3389/fgene.2014.00332 PMC4181244

[b47] Yuan J. . Genome-wide association studies for feed intake and efficiency in two laying periods of chickens. Genet. Sel. Evol. 47, 82 (2015). 26475174 10.1186/s12711-015-0161-1 PMC4608132

[b48] Yang J. . Genomic inflation factors under polygenic inheritance. Eur. J. Hum. Genet. 19, 807–812 (2011). 21407268 10.1038/ejhg.2011.39 PMC3137506

[b49] Tuiskula-Haavisto M. . Mapping of quantitative trait loci affecting quality and production traits in egg layers. Poult. Sci. 81, 919–27 (2002). 12162350 10.1093/ps/81.7.919

[b50] Tuiskula-Haavisto M. . Quantitative trait loci with parent-of-origin effects in chicken. Genet. Res. 84, 57–66 (2004). 15663259 10.1017/s0016672304006950

[b51] Rosário M. F. do, Ledur M. C., Moura A. S. A. M. T., Coutinho L. L. & Garcia A. A. F. Genotypic characterization of microsatellite markers in broiler and layer selected chicken lines and their reciprocal F1s. Sci. Agric. 66, 150–158 (2009).

[b52] Nassar M. K., Goraga Z. S. & Brockmann G. A. Quantitative trait loci segregating in crosses between New Hampshire and White Leghorn chicken lines: IV. Growth performance. Anim. Genet. 46, 441–446 (2015). 25908024 10.1111/age.12298

[b53] Goraga Z. S., Nassar M. K. & Brockmann G. A. Quantitative trait loci segregating in crosses between New Hampshire and White Leghorn chicken lines: I. egg production traits. Anim. Genet. 43, 183–189 (2012). 22404354 10.1111/j.1365-2052.2011.02233.x

[b54] Gu X. . Genome-Wide Association Study of Body Weight in Chicken F2 Resource Population. PLoS One 6, e21872 (2011). 21779344 10.1371/journal.pone.0021872 PMC3136483

[b55] Ledur M. C., Navarro N. & Pérez-Enciso M. Large-scale SNP genotyping in crosses between outbred lines: how useful is it? Heredity (Edinb). 105, 173–182 (2010). 19844266 10.1038/hdy.2009.149

[b56] Liu X. . Mapping quantitative trait loci affecting body weight and abdominal fat weight on chicken chromosome one. Poult. Sci. 86, 1084–9 (2007). 17495077 10.1093/ps/86.6.1084

[b57] Hansen C. . Identification of QTL for production traits in chickens. Anim. Biotechnol. 16, 67–79 (2005). 15926264 10.1081/abio-200055016

[b58] Rabie T. S. K. M. . Genetic mapping of quantitative trait loci affecting susceptibility in chicken to develop pulmonary hypertension syndrome. Anim. Genet. 36, 468–76 (2005). 16293119 10.1111/j.1365-2052.2005.01346.x

[b59] Ankra-Badu G. a. . Mapping QTL for growth and shank traits in chickens divergently selected for high or low body weight. Anim. Genet. 41, 400–5 (2010). 20096032 10.1111/j.1365-2052.2009.02017.x

[b60] Liu R. . Genome-Wide Association Study Identifies Loci and Candidate Genes for Body Composition and Meat Quality Traits in Beijing-You Chickens. PLoS One 8, e61172 (2013). 23637794 10.1371/journal.pone.0061172 PMC3630158

[b61] Abasht B. & Lamont S. J. Genome-wide association analysis reveals cryptic alleles as an important factor in heterosis for fatness in chicken F2 population. Anim. Genet. 38, 491–8 (2007). 17894563 10.1111/j.1365-2052.2007.01642.x

[b62] Liu W. . A genome-wide SNP scan reveals novel loci for egg production and quality traits in white leghorn and brown-egg dwarf layers. PLoS One 6, e28600 (2011). 22174844 10.1371/journal.pone.0028600 PMC3234275

[b63] Baron E. E. . QTL for percentage of carcass and carcass parts in a broiler x layer cross. Anim. Genet. 42, 117–124 (2011). 20880336 10.1111/j.1365-2052.2010.02105.x

[b64] Podisi B. K., Knott S. A., Burt D. W. & Hocking P. M. Comparative analysis of quantitative trait loci for body weight, growth rate and growth curve parameters from 3 to 72 weeks of age in female chickens of a broiler–layer cross. BMC Genet. 14, 22 (2013). 23496818 10.1186/1471-2156-14-22 PMC3606837

[b65] Podisi B. K. . Overlap of quantitative trait loci for early growth rate, and for body weight and age at onset of sexual maturity in chickens. Reproduction 141, 381–389 (2011). 21177954 10.1530/REP-10-0276

[b66] Sewalem A. . Mapping of quantitative trait loci for body weight at three, six, and nine weeks of age in a broiler layer cross. Poult. Sci. 81, 1775–81 (2002). 12512565 10.1093/ps/81.12.1775

[b67] Carlborg O., Hocking P. M., Burt D. W. & Haley C. S. Simultaneous mapping of epistatic QTL in chickens reveals clusters of QTL pairs with similar genetic effects on growth. Genet. Res. 83, 197–209 (2004). 15462413 10.1017/s0016672304006779

[b68] Eck S. H. . Whole genome sequencing of a single Bos taurus animal for single nucleotide polymorphism discovery. Genome Biol. 10, R82 (2009). 19660108 10.1186/gb-2009-10-8-r82 PMC2745763

[b69] Kang H. M. . Efficient Control of Population Structure in Model Organism Association Mapping. Genetics 178, 1709–1723 (2008). 18385116 10.1534/genetics.107.080101 PMC2278096

[b70] Misztal I. . BLUPF90 and related programs (BGF90). Proc. 7th World Congr. Genet. Appl. to Livest. Prod. 28, 21–22 (2002).

[b71] Meyer K. & Houle D. Sampling based approximation of confidence intervals for functions of genetic covariance matrices. Proc. Assoc. Advmt. Anim. Breed. Genet. 20, 523–526 (2013).

[b72] Nicodemus K. K., Liu W., Chase G. a., Tsai Y.-Y. & Fallin M. D. Comparison of type I error for multiple test corrections in large single-nucleotide polymorphism studies using principal components versus haplotype blocking algorithms. BMC Genet. 6, S78 (2005). 16451692 10.1186/1471-2156-6-S1-S78 PMC1866703

[b73] Barrett J. C., Fry B., Maller J. & Daly M. J. Haploview: Analysis and visualization of LD and haplotype maps. Bioinformatics 21, 263–265 (2005). 15297300 10.1093/bioinformatics/bth457

