# Supplementary material for: Genome-wide association study for performance traits in chickens using genotype by sequencing approach
Source: Sci Rep. 2017 Feb 9;7:41748. doi: 10.1038/srep41748 (PMC5299454; doi:10.1038/srep41748)
Supplement: Supplementary Information [file srep41748-s1.doc]

**Genome-wide association study for performance traits in chickens using genotype by sequencing approach**

Fábio Pértille^1^; Gabriel Costa Monteiro Moreira^1^, Ricardo Zanella^2^, José de Ribamar da Silva Nunes^1^, Clarisa Boschiero^1^; Gregori Rovadoscki^1^; Gerson Barreto Mourão^1^, Mônica Corrêa Ledur^3^ & Luiz Lehmann Coutinho^1*^

^1^ Animal Biotechnology Laboratory, Animal Science and Pastures Department, University of São Paulo (USP)/ Luiz de Queiroz College of Agriculture (ESALQ), Piracicaba, São Paulo, Brazil

^2^College of Agronomy and Veterinary Medicine, Veterinary School, University of Passo Fundo, Rio Grande do Sul, Brazil

^3^ Embrapa Suínos e Aves, Concórdia, Santa Catarina, Brazil

*Corresponding author:

Email: [llcoutinho@usp.br](mailto:llcoutinho@usp.br) (LLC)

**SUPPLEMENTARY INFORMATION**

**1. Supplementary figures**

Supplementary Fig. S1. Suggestively associated SNPs with feed intake (a), and feed efficiency (b) both adjusted for body weight at 35 days; body weight at 41 (c) days of age and birth weight (d) are presented by Manhattan (left side) and QQ (right side) plots. The y-axis is shown as -log10 (p-value) for both graphs. On the left, the blue line indicates suggestive genome-wise association (P < 1.57E-05) with the respective trait. On the right side, the QQ-plots show the relation of normal theoretical quantiles of the probability distributions between expected (x-axis) and observed (y-axis) p-values from each respective associated trait.

Supplementary Fig. S2. Haplotype blocks obtained by the solid spine of LD and family structure using Haploview 4.2. The header represents the block number and tagSNP for each 17 blocks obtained from 94 markers in LD presented in Figure 3 of the manuscript (r^2^>0.6 except block 17, r^2^>0.2) in 444 F_2_ individuals (a). The other letters represent the frequency of the F_2_ blocks in the 8 F_1_ (b), 5 CC maternal parental line (c) and, 5 TT paternal parental line (d).

Supplementary Fig. S3. Figure 4. Karyotype of the QTLs (from Animal QTLdb) distribution regions of the chicken genome overlapping suggestive and significant SNPs associated with performance traits (black marks). We subset only QTLs mapped for birth weight (BW1), body weight at 35 (BW35) and 41 (BW41) days of age, and feed intake (f.intake) traits performed in the same F_2_ population used in the presented manuscript and published before.

**2. Supplementary tables**

Supplementary Table S1. Mendelian descriptions for suggestive (P < 1.57E-05) and/or genome-wise associated (P < 7.86E-07) 94 markers from 444 F_2_ individuals.

Supplementary Table S2. The table is rating who has the advantageous alleles from the parental lines (mother/father) (adv. parental) for the trait evaluated from each of the 17 LD blocks. The first column indicates the block number, the second and third indicate whether this haplotype is fixed in the father/mother and the fourth column indicates the parental which concentrates more frequently the advantageous allele.

Supplementary Table S3. List of 253 QTLs overlapping 81 markers obtained from overlapping test of 94 associated (P < 7.86E-07) and/or suggestive (P < 1.57E-05) associated markers from 444 F_2_ individuals in this study against 1,458 QTLs obtained from the Animal QTLdb.

Supplementary Table S4. Functional annotation of 94 associated (P < 7.86E-07) and/or suggestive associated (P < 1.57E-05) SNPs from 444 F_2_ individuals using Variant Effect Predictor (VEP) tool v.71 online ^1^. The last column is the classification of the SNPs as overlapping QTLs (TRUE) or not overlapping QTLs (FALSE). These QTLs were the same presented in Table S3 online.

Supplementary Table S5. List of 40 QTLs mapped using microsatellite markers, from the same F_2_ population used in this study. This information was filtered by the Chicken Animal QTLdb.

**Supplementary Spreadsheets**

Supplementary Spreadsheet S1. Association SNPs stats for each of the 94 markers with genome-wise significance (P < 7.86E-07) in pink highlight color and suggestive genome-wise association (P < 1.57E-05) to the others with the respective trait described in column M. Where: eff.adj35 (feed efficiency), f.conv.adj35 (feed conversion), f.int.adj35 (feed intake), all between 35 and 41 days of age and adjusted for body weight at 35 days; bw35, bw41 is body weight at 35 and 41 days of age, respectively, and birthW is birth weight. Markers overlapping with QTL (TRUE) or not overlapping with QTL (FALSE) regions are indicated in column T.

**1. Supplementary figures**

Supplementary Fig. S1.

(a)


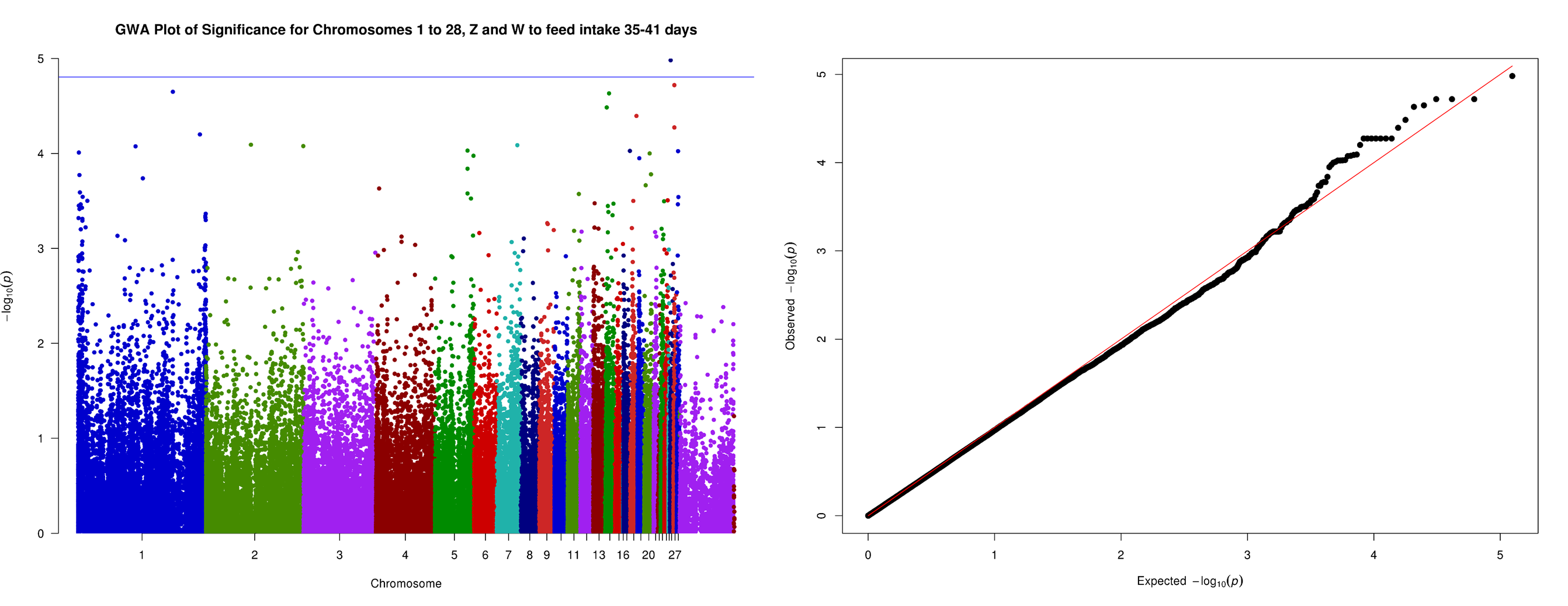


(b)


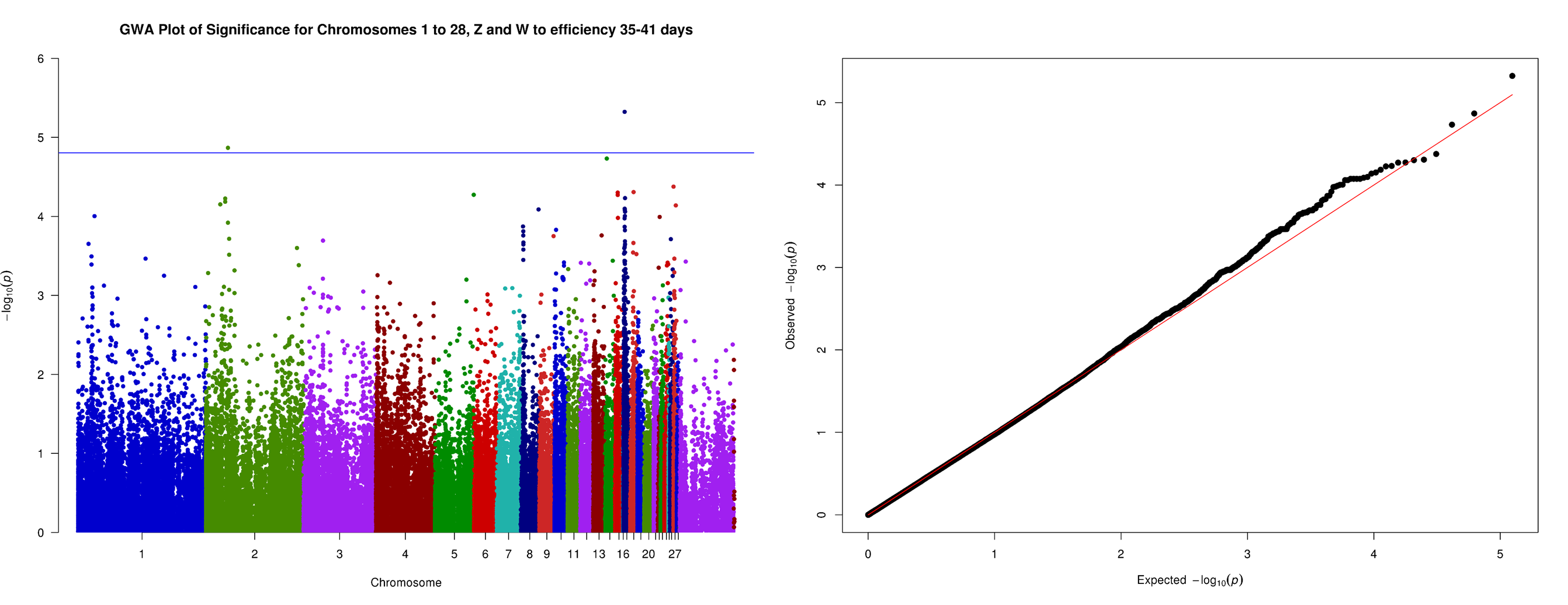


(c)


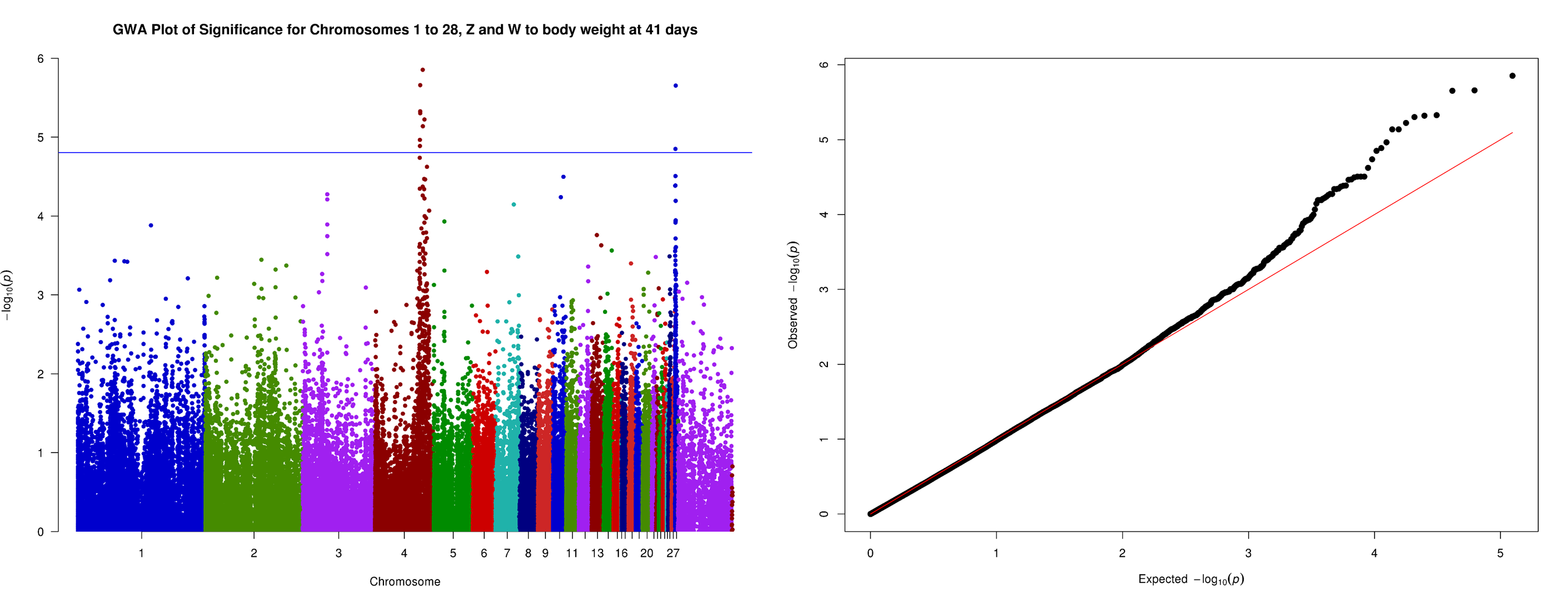


(d)


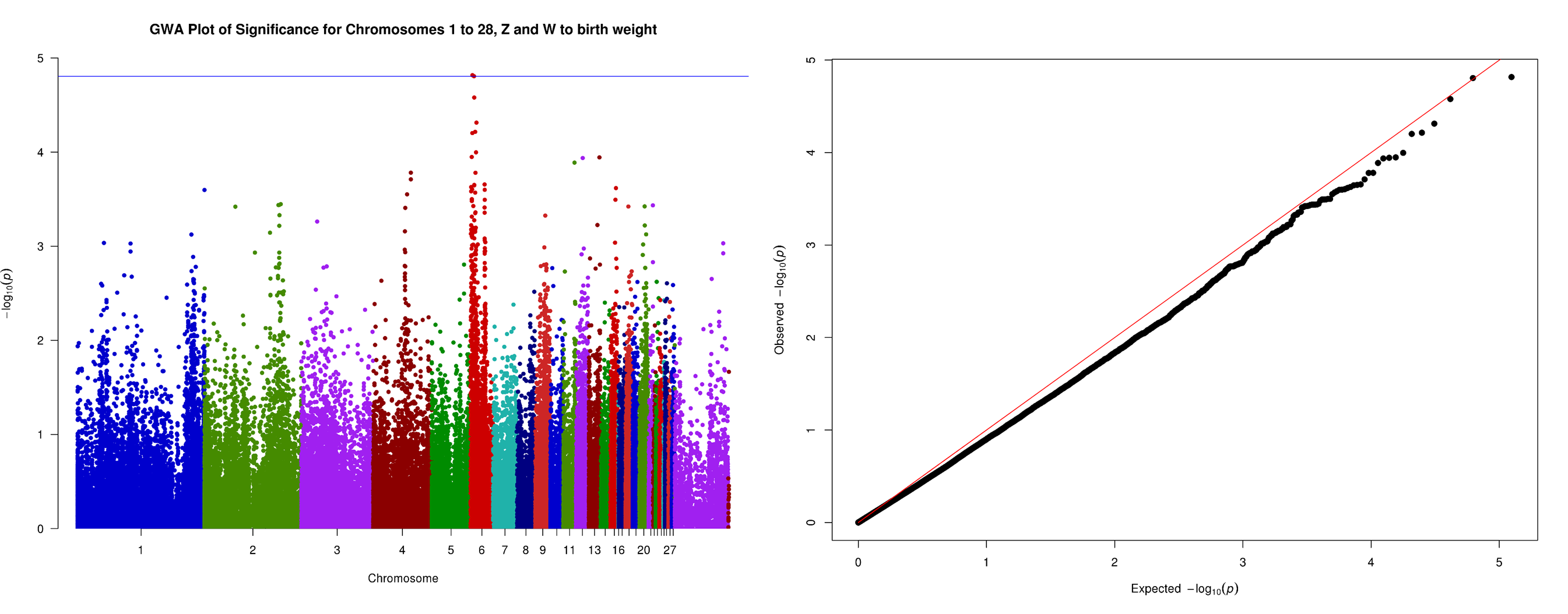


Supplementary Fig. S2.


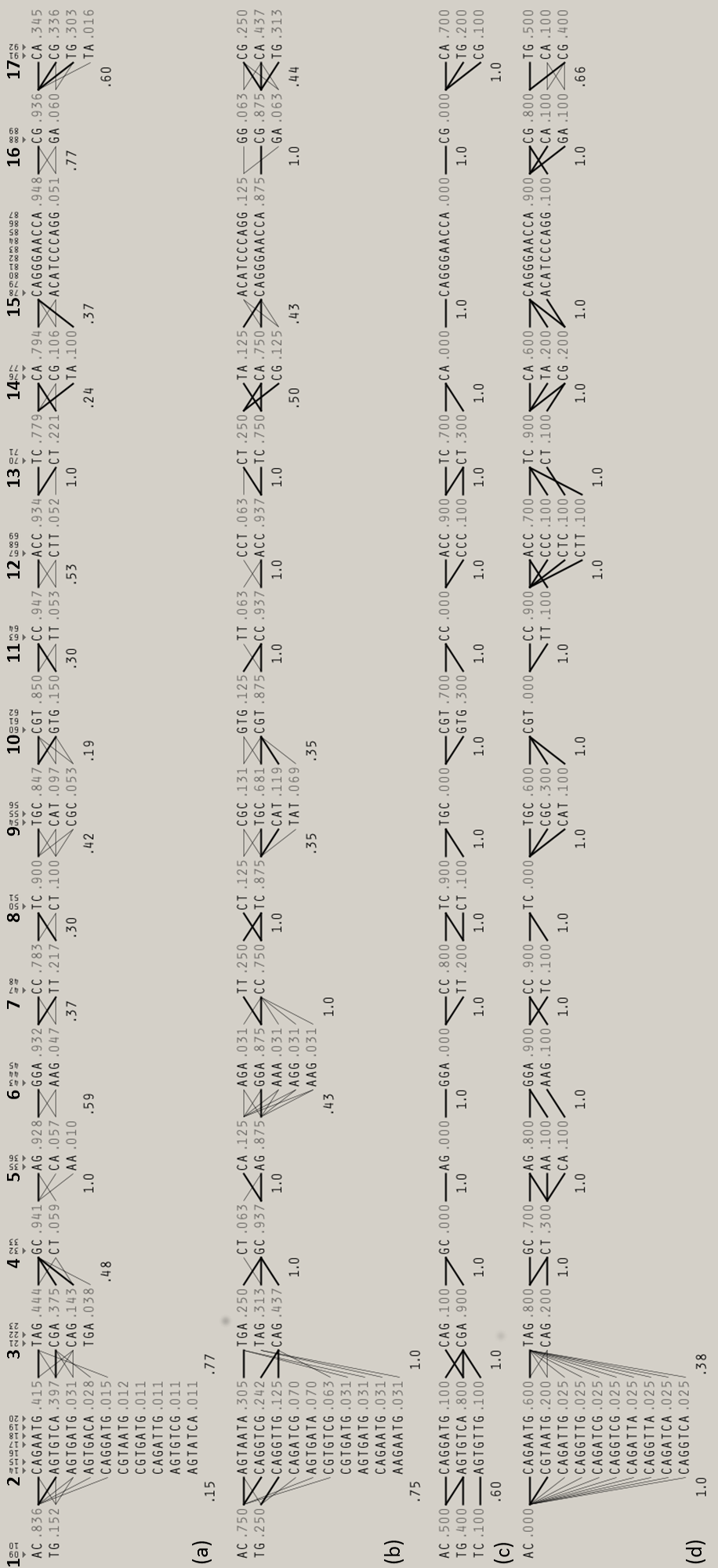


Supplementary Fig. S3.

**
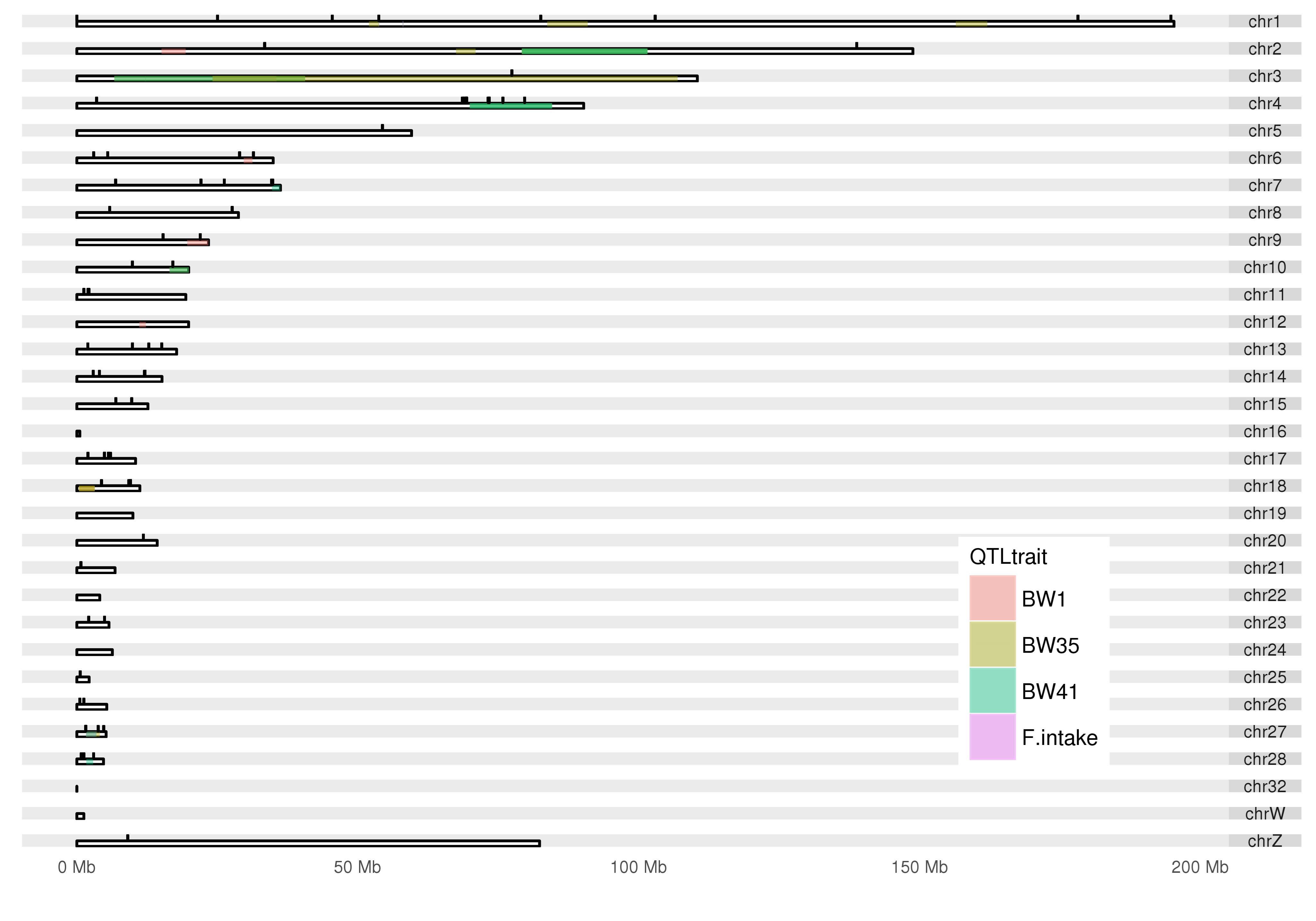
**

**2. Supplementary tables**

Supplementary Table S1.

| **#** | **Markers**  **Chr_pos** | **ObsHET** | **PredHET** | **HWpval** |  | **MAF** | **Alleles** |
| --- | --- | --- | --- | --- | --- | --- | --- |
| 1 | 1_3706 | 0.21 | 0.19 | 4.71E-02 |  | 0.11 | C:G |
| 2 | 1_25041791 | 0.22 | 0.20 | 1.21E-01 |  | 0.11 | C:T |
| 3 | 1_45468342 | 0.12 | 0.11 | 1.00E+00 |  | 0.06 | T:C |
| 4 | 1_53749693 | 0.10 | 0.10 | 1.00E+00 |  | 0.05 | G:A |
| 5 | 1_82575973 | 0.53 | 0.39 | 7.40E-18 | * | 0.27 | T:C |
| 6 | 1_102944294 | 0.24 | 0.22 | 3.81E-02 |  | 0.13 | A:C |
| 7 | 1_178184151 | 0.12 | 0.12 | 9.36E-01 |  | 0.06 | G:A |
| 8 | 1_194726625 | 0.21 | 0.19 | 5.82E-02 |  | 0.11 | A:C |
| 9 | 2_33436237 | 0.30 | 0.27 | 1.25E-02 |  | 0.16 | A:T |
| 10 | 2_33437336 | 0.31 | 0.27 | 5.87E-05 | * | 0.16 | C:G |
| 11 | 2_138815862 | 0.09 | 0.10 | 5.43E-02 |  | 0.05 | G:A |
| 12 | 3_77435334 | 0.13 | 0.12 | 8.16E-01 |  | 0.07 | C:T |
| 13 | 4_3514658 | 0.17 | 0.16 | 2.90E-01 |  | 0.09 | G:T |
| 14 | 4_68623163 | 0.52 | 0.50 | 5.79E-01 |  | 0.49 | A:C |
| 15 | 4_68882750 | 0.48 | 0.50 | 4.25E-01 |  | 0.47 | G:A |
| 16 | 4_68882765 | 0.48 | 0.50 | 3.69E-01 |  | 0.47 | T:G |
| 17 | 4_69297525 | 0.43 | 0.50 | 6.80E-03 |  | 0.47 | G:A |
| 18 | 4_69370079 | 0.43 | 0.50 | 4.20E-03 |  | 0.46 | A:T |
| 19 | 4_69372005 | 0.49 | 0.50 | 7.21E-01 |  | 0.48 | T:C |
| 20 | 4_69372065 | 0.49 | 0.50 | 9.01E-01 |  | 0.47 | G:A |
| 21 | 4_73210325 | 0.42 | 0.50 | 9.00E-04 | * | 0.48 | C:T |
| 22 | 4_73407243 | 0.46 | 0.49 | 2.58E-01 |  | 0.41 | A:G |
| 23 | 4_73407249 | 0.46 | 0.49 | 2.58E-01 |  | 0.41 | G:A |
| 24 | 4_75842191 | 0.42 | 0.49 | 7.00E-03 |  | 0.41 | A:C |
| 25 | 4_79707389 | 0.37 | 0.45 | 8.06E-05 | * | 0.35 | C:G |
| 26 | 5_54401932 | 0.19 | 0.18 | 1.16E-01 |  | 0.10 | C:T |
| 27 | 6_2997851 | 0.14 | 0.17 | 1.60E-03 |  | 0.10 | G:A |
| 28 | 6_5492179 | 0.49 | 0.50 | 8.21E-01 |  | 0.47 | A:C |
| 29 | 6_28967516 | 0.29 | 0.25 | 4.00E-04 | * | 0.15 | G:T |
| 30 | 6_31436207 | 0.41 | 0.33 | 1.58E-09 | * | 0.21 | T:C |
| 31 | 7_6904443 | 0.94 | 0.50 | 2.00E-97 | * | 0.47 | G:A |
| 32 | 7_22092999 | 0.11 | 0.11 | 1.00E+00 |  | 0.06 | G:C |
| 33 | 7_22093009 | 0.11 | 0.11 | 1.00E+00 |  | 0.06 | C:T |
| 34 | 7_26252164 | 0.13 | 0.13 | 7.40E-01 |  | 0.07 | C:T |
| 35 | 7_34668134 | 0.11 | 0.12 | 1.54E-01 |  | 0.06 | A:C |
| 36 | 7_34855716 | 0.13 | 0.13 | 7.78E-01 |  | 0.07 | G:A |
| 37 | 8_5848411 | 0.30 | 0.26 | 3.00E-04 | * | 0.15 | T:C |
| 38 | 8_27651130 | 0.15 | 0.15 | 9.07E-01 |  | 0.08 | T:C |
| 39 | 9_15349595 | 0.12 | 0.12 | 9.36E-01 |  | 0.06 | G:A |
| 40 | 9_21971358 | 0.21 | 0.19 | 5.24E-02 |  | 0.11 | C:T |
| 41 | 10_9890328 | 0.33 | 0.28 | 1.46E-05 | * | 0.17 | A:G |
| 42 | 10_17086845 | 0.12 | 0.12 | 9.36E-01 |  | 0.06 | G:A |
| 43 | 11_1249418 | 0.11 | 0.10 | 1.00E+00 |  | 0.06 | G:A |
| 44 | 11_1997302 | 0.12 | 0.11 | 1.00E+00 |  | 0.06 | G:A |
| 45 | 11_2149223 | 0.12 | 0.11 | 1.00E+00 |  | 0.06 | A:G |
| 46 | 13_1958055 | 0.28 | 0.25 | 6.00E-04 | * | 0.14 | C:T |
| 47 | 13_9894951 | 0.43 | 0.34 | 1.43E-10 | * | 0.22 | C:T |
| 48 | 13_9894952 | 0.43 | 0.34 | 1.43E-10 | * | 0.22 | C:T |
| 49 | 13_12804655 | 0.34 | 0.31 | 2.00E-02 |  | 0.19 | T:C |
| 50 | 13_15109350 | 0.20 | 0.18 | 9.60E-02 |  | 0.10 | T:C |
| 51 | 13_15109354 | 0.20 | 0.18 | 9.60E-02 |  | 0.10 | C:T |
| 52 | 14_2918142 | 0.22 | 0.20 | 2.74E-01 |  | 0.12 | C:A |
| 53 | 14_4019669 | 0.26 | 0.23 | 2.50E-03 |  | 0.13 | G:T |
| 54 | 14_12043802 | 0.29 | 0.26 | 1.90E-03 |  | 0.15 | T:C |
| 55 | 14_12127914 | 0.20 | 0.18 | 9.60E-02 |  | 0.10 | G:A |
| 56 | 14_12127934 | 0.20 | 0.18 | 9.60E-02 |  | 0.10 | C:T |
| 57 | 15_6942668 | 0.55 | 0.40 | 1.22E-19 | * | 0.28 | G:A |
| 58 | 15_9763685 | 0.28 | 0.25 | 7.00E-04 | * | 0.14 | C:A |
| 59 | 17_1976385 | 0.30 | 0.42 | 4.25E-09 | * | 0.30 | C:G |
| 60 | 17_4917070 | 0.30 | 0.26 | 3.00E-04 | * | 0.15 | C:G |
| 61 | 17_4917071 | 0.30 | 0.26 | 3.00E-04 | * | 0.15 | G:T |
| 62 | 17_4917072 | 0.30 | 0.26 | 3.00E-04 | * | 0.15 | T:G |
| 63 | 17_5621341 | 0.10 | 0.10 | 1.00E+00 |  | 0.05 | C:T |
| 64 | 17_5621374 | 0.10 | 0.10 | 1.00E+00 |  | 0.05 | C:T |
| 65 | 17_5986188 | 0.56 | 0.41 | 1.79E-20 | * | 0.28 | C:T |
| 66 | 18_4390068 | 0.10 | 0.10 | 7.77E-01 |  | 0.06 | C:T |
| 67 | 18_9253828 | 0.12 | 0.11 | 1.00E+00 |  | 0.06 | A:C |
| 68 | 18_9551090 | 0.11 | 0.11 | 1.00E+00 |  | 0.06 | C:T |
| 69 | 18_9554600 | 0.11 | 0.11 | 9.82E-01 |  | 0.06 | C:T |
| 70 | 20_11847031 | 0.44 | 0.34 | 5.59E-11 | * | 0.22 | T:C |
| 71 | 20_11847038 | 0.44 | 0.34 | 5.59E-11 | * | 0.22 | C:T |
| 72 | 21_725260 | 0.22 | 0.20 | 3.02E-02 |  | 0.11 | G:A |
| 73 | 23_2109713 | 0.23 | 0.21 | 1.47E-02 |  | 0.12 | G:A |
| 74 | 23_4929597 | 0.19 | 0.18 | 2.86E-01 |  | 0.10 | A:G |
| 75 | 25_601833 | 0.31 | 0.27 | 7.10E-05 | * | 0.16 | T:C |
| 76 | 26_534090 | 0.18 | 0.18 | 1.00E+00 |  | 0.10 | C:T |
| 77 | 26_1249195 | 0.19 | 0.19 | 1.00E+00 |  | 0.11 | A:G |
| 78 | 27_1587217 | 0.10 | 0.10 | 1.00E+00 |  | 0.05 | C:A |
| 79 | 27_1587218 | 0.10 | 0.10 | 1.00E+00 |  | 0.05 | A:C |
| 80 | 27_1587219 | 0.10 | 0.10 | 1.00E+00 |  | 0.05 | G:A |
| 81 | 27_1587220 | 0.10 | 0.10 | 1.00E+00 |  | 0.05 | G:T |
| 82 | 27_1587221 | 0.10 | 0.10 | 1.00E+00 |  | 0.05 | G:C |
| 83 | 27_1587222 | 0.10 | 0.10 | 1.00E+00 |  | 0.05 | A:C |
| 84 | 27_1587224 | 0.10 | 0.10 | 1.00E+00 |  | 0.05 | A:C |
| 85 | 27_1587226 | 0.10 | 0.10 | 1.00E+00 |  | 0.05 | C:A |
| 86 | 27_1587227 | 0.10 | 0.10 | 1.00E+00 |  | 0.05 | C:G |
| 87 | 27_1587228 | 0.10 | 0.10 | 1.00E+00 |  | 0.05 | A:G |
| 88 | 27_3798382 | 0.12 | 0.12 | 1.00E+00 |  | 0.06 | C:G |
| 89 | 27_3798470 | 0.12 | 0.11 | 1.00E+00 |  | 0.06 | G:A |
| 90 | 27_4784671 | 0.38 | 0.31 | 9.31E-08 | * | 0.19 | G:A |
| 91 | 28_788219 | 0.40 | 0.43 | 1.02E-01 |  | 0.32 | C:T |
| 92 | 28_1252731 | 0.51 | 0.46 | 5.19E-02 |  | 0.36 | G:A |
| 93 | 28_2986566 | 0.33 | 0.28 | 1.79E-05 | * | 0.17 | G:A |
| 94 | Z_9064309 | 0.21 | 0.19 | 1.75E-01 |  | 0.11 | A:G |

*HW p-value cutoff: 0.001, Chr – chromossome, pos – marker position on chromosome.

Supplementary Table. S2.

| **Block** | **Father** | **Mother** | **adv. parental** |
| --- | --- | --- | --- |
| 1 | fixed | variable | father |
| 2 | variable | variable | father |
| 3 | fixed | variable | father |
| 4 | variable | fixed | mother |
| 5 | variable | fixed | mother |
| 6 | variable | fixed | mother |
| 7 | fixed | variable | father |
| 8 | fixed | variable | father |
| 9 | variable | fixed | mother |
| 10 | fixed | variable | father |
| 11 | variable | fixed | mother |
| 12 | variable | fixed | mother |
| 13 | variable | variable | father |
| 14 | variable | fixed | mother |
| 15 | variable | fixed | mother |
| 16 | variable | fixed | mother |
| 17 | variable | variable | father/mother |

Supplementary Table S3.

| **chr** | **start** | **end** | **QTL_ID** | **Trait** | **Abrev** |
| --- | --- | --- | --- | --- | --- |
| chr1 | 2329958 | 64931354 | QTL_ID=9406 | Body_weight_(63_days) | bw |
| chr1 | 7010437 | 27734493 | QTL_ID=6752 | Body_weight_(70_days) | bw |
| chr1 | 23286118 | 25486484 | QTL_ID=6751 | Body_weight_(63_days) | bw |
| chr1 | 23286118 | 25486484 | QTL_ID=6753 | Body_weight_(77_days) | bw |
| chr1 | 25275933 | 48175152 | QTL_ID=1775 | Body_weight | bw |
| chr1 | 25275933 | 48175152 | QTL_ID=9739 | Body_weight_(21_days) | bw |
| chr1 | 25275933 | 48175152 | QTL_ID=9740 | Body_weight_(42_days) | bw |
| chr1 | 25275933 | 48175152 | QTL_ID=9741 | Body_weight_(63_days) | bw |
| chr1 | 28436329 | 63038738 | QTL_ID=1807 | Body_weight | bw |
| chr1 | 28436329 | 63038738 | QTL_ID=1808 | Body_weight | bw |
| chr1 | 33174045 | 134863919 | QTL_ID=17076 | Body_weight_(140_days) | bw |
| chr1 | 33279320 | 47367912 | QTL_ID=6675 | Feed_conversion_ratio | f.conv |
| chr1 | 33279320 | 47367912 | QTL_ID=6674 | Residual_feed_intake | f.intake |
| chr1 | 42474406 | 52428237 | QTL_ID=1788 | Body_weight | bw |
| chr1 | 45111848 | 45996329 | QTL_ID=24950 | Body_weight | bw |
| chr1 | 50511472 | 82050274 | QTL_ID=55913 | Body_weight_(105_days) | bw |
| chr1 | 50511472 | 71471379 | QTL_ID=55933 | Growth_(35-70_days) | grow |
| chr1 | 50722023 | 68558759 | QTL_ID=55902 | Body_weight_(35_days) | bw |
| chr1 | 50722023 | 69541330 | QTL_ID=55908 | Body_weight_(70_days) | bw |
| chr1 | 50722023 | 67611281 | QTL_ID=55927 | Growth_(0-35_days) | grow |
| chr1 | 51977644 | 195276750 | QTL_ID=1797 | Body_weight | bw |
| chr1 | 51977644 | 55261640 | QTL_ID=24872 | Body_weight_(168_days) | bw |
| chr1 | 51977644 | 55261640 | QTL_ID=24839 | Body_weight_(21_days) | bw |
| chr1 | 51977644 | 53798691 | ***QTL_ID=3324** | Body_weight_(35_days) | bw |
| chr1 | 51977644 | 55261640 | QTL_ID=24848 | Body_weight_(42_days) | bw |
| chr1 | 51977644 | 53798691 | QTL_ID=3325 | Body_weight_(42_days) | bw |
| chr1 | 51977644 | 55261640 | QTL_ID=14462 | Body_weight_(day_of_f.intakerst_egg) | bw |
| chr1 | 51977644 | 55261640 | QTL_ID=14467 | Body_weight_(day_of_f.intakerst_egg) | bw |
| chr1 | 57051322 | 152887033 | QTL_ID=55937 | Growth_(70-105_days) | grow |
| chr1 | 59332289 | 134126991 | QTL_ID=55919 | Body_weight_(140_days) | bw |
| chr1 | 81804631 | 84531095 | QTL_ID=9110 | Growth_(post-challenge) | grow |
| chr1 | 90354029 | 123032393 | QTL_ID=1821 | Feed_efficience | effic |
| chr1 | 92255503 | 108492449 | QTL_ID=6813 | Body_weight_(56_days) | bw |
| chr1 | 92255503 | 108492449 | QTL_ID=6814 | Body_weight_(56_days) | bw |
| chr1 | 92255503 | 108492449 | QTL_ID=6815 | Body_weight_(63_days) | bw |
| chr1 | 92255503 | 108492449 | QTL_ID=6812 | Body_weight_(7_days) | bw |
| chr1 | 92255503 | 108492449 | QTL_ID=6816 | Body_weight_(77_days) | bw |
| chr1 | 92255503 | 108492449 | QTL_ID=6817 | Body_weight_(84_days) | bw |
| chr1 | 94157976 | 113198023 | QTL_ID=1822 | Feed_intake | f.intake |
| chr1 | 143677314 | 191778894 | QTL_ID=6583 | Body_weight_(112_days) | bw |
| chr1 | 143677314 | 191778894 | QTL_ID=6584 | Body_weight_(200_days) | bw |
| chr1 | 143677314 | 191778894 | QTL_ID=6582 | Body_weight_(46_days) | bw |
| chr1 | 143677314 | 191778894 | QTL_ID=6579 | Body_weight_(8_days) | bw |
| chr1 | 143677314 | 191778894 | QTL_ID=6588 | Growth_(112-200_days) | grow |
| chr1 | 143677314 | 191778894 | QTL_ID=6585 | Growth_(1-8_days) | grow |
| chr1 | 143677314 | 191778894 | QTL_ID=6587 | Growth_(46-112_days) | grow |
| chr1 | 143677314 | 191778894 | QTL_ID=6586 | Growth_(8-46_days) | grow |
| chr1 | 156472083 | 191778894 | QTL_ID=1855 | Body_weight | bw |
| chr1 | 156472083 | 191778894 | QTL_ID=1858 | Body_weight | bw |
| chr1 | 156472083 | 195276750 | QTL_ID=24873 | Body_weight_(168_days) | bw |
| chr1 | 156472083 | 191778894 | QTL_ID=9750 | Body_weight_(21_days) | bw |
| chr1 | 156472083 | 191778894 | QTL_ID=9751 | Body_weight_(42_days) | bw |
| chr1 | 178182301 | 178252485 | QTL_ID=16642 | Body_weight_(28_days) | bw |
| chr1 | 178182301 | 178252485 | QTL_ID=16667 | Body_weight_(28_days) | bw |
| chr1 | 178182301 | 178252485 | QTL_ID=16643 | Body_weight_(35_days) | bw |
| chr1 | 178182301 | 178252485 | QTL_ID=16644 | Body_weight_(42_days) | bw |
| chr1 | 178182301 | 178252485 | QTL_ID=16669 | Body_weight_(42_days) | bw |
| chr1 | 178182301 | 178252485 | QTL_ID=16645 | Body_weight_(49_days) | bw |
| chr1 | 178182301 | 178252485 | QTL_ID=16670 | Body_weight_(49_days) | bw |
| chr1 | 178182301 | 178252485 | QTL_ID=16646 | Body_weight_(56_days) | bw |
| chr1 | 178182301 | 178252485 | QTL_ID=16671 | Body_weight_(56_days) | bw |
| chr1 | 178182301 | 178252485 | QTL_ID=16647 | Body_weight_(63_days) | bw |
| chr1 | 178182301 | 178252485 | QTL_ID=16672 | Body_weight_(63_days) | bw |
| chr1 | 178182301 | 178252485 | QTL_ID=16648 | Body_weight_(70_days) | bw |
| chr1 | 178182301 | 178252485 | QTL_ID=16673 | Body_weight_(70_days) | bw |
| chr1 | 178182301 | 178252485 | QTL_ID=16649 | Body_weight_(77_days) | bw |
| chr1 | 178182301 | 178252485 | QTL_ID=16674 | Body_weight_(77_days) | bw |
| chr1 | 178182301 | 178252485 | QTL_ID=16650 | Body_weight_(84_days) | bw |
| chr1 | 178182301 | 178252485 | QTL_ID=16675 | Body_weight_(84_days) | bw |
| chr2 | 578373 | 37938317 | QTL_ID=1871 | Body_weight | bw |
| chr2 | 578373 | 37938317 | QTL_ID=1872 | Body_weight | bw |
| chr2 | 2452503 | 37938317 | QTL_ID=1873 | Body_weight | bw |
| chr2 | 15053040 | 37938317 | QTL_ID=1874 | Body_weight | bw |
| chr2 | 17958970 | 37831227 | QTL_ID=6848 | Body_weight_(42_days) | bw |
| chr2 | 21441194 | 71328860 | QTL_ID=55934 | Growth_(35-70_days) | grow |
| chr2 | 29064142 | 108449411 | QTL_ID=55909 | Body_weight_(70_days) | bw |
| chr2 | 29345262 | 65036736 | QTL_ID=55914 | Body_weight_(105_days) | bw |
| chr2 | 103056068 | 144263101 | QTL_ID=9415 | Body_weight_(63_days) | bw |
| chr2 | 129556328 | 148809762 | QTL_ID=1928 | Body_weight_(test_end) | bw |
| chr3 | 2811981 | 103804757 | QTL_ID=1979 | Body_weight | bw |
| chr3 | 2811981 | 103804757 | QTL_ID=1980 | Body_weight | bw |
| chr3 | 6645961 | 106970113 | ***QTL_ID=7180** | Body_weight_(35_days) | bw |
| chr3 | 6841859 | 102661494 | QTL_ID=55904 | Body_weight_(35_days) | bw |
| chr3 | 6841859 | 99827114 | QTL_ID=55929 | Growth_(0-35_days) | grow |
| chr3 | 24160710 | 79800194 | QTL_ID=1957 | Body_weight | bw |
| chr3 | 37579996 | 108174898 | QTL_ID=1961 | Body_weight | bw |
| chr3 | 37579996 | 108174898 | QTL_ID=1962 | Body_weight | bw |
| chr3 | 45203763 | 84080722 | QTL_ID=6611 | Body_weight_(112_days) | bw |
| chr3 | 45203763 | 84080722 | QTL_ID=6612 | Body_weight_(200_days) | bw |
| chr3 | 45203763 | 84080722 | QTL_ID=6610 | Body_weight_(8_days) | bw |
| chr3 | 45203763 | 84080722 | QTL_ID=6613 | Growth_(1-8_days) | grow |
| chr3 | 45311696 | 104141066 | QTL_ID=9420 | Body_weight_(63_days) | bw |
| chr3 | 57793506 | 84765752 | QTL_ID=11768 | Body_weight_(49_days) | bw |
| chr3 | 57793506 | 84765752 | QTL_ID=11772 | Body_weight_(63_days) | bw |
| chr3 | 58466018 | 88819995 | QTL_ID=1969 | Body_weight | bw |
| chr3 | 58466018 | 88819995 | QTL_ID=1972 | Body_weight | bw |
| chr3 | 72032411 | 84765752 | QTL_ID=9127 | Growth_(post-challenge) | grow |
| chr4 | 3459678 | 19246773 | QTL_ID=1989 | Body_weight | bw |
| chr4 | 3459678 | 19246773 | QTL_ID=1990 | Body_weight | bw |
| chr4 | 3459678 | 19246773 | QTL_ID=1991 | Body_weight | bw |
| chr4 | 3459678 | 16468784 | QTL_ID=24854 | Body_weight_(42_days) | bw |
| chr4 | 3459678 | 16468784 | QTL_ID=24865 | Body_weight_(84_days) | bw |
| chr4 | 17039416 | 80254980 | QTL_ID=24875 | Body_weight_(168_days) | bw |
| chr4 | 17039416 | 80254980 | QTL_ID=24842 | Body_weight_(21_days) | bw |
| chr4 | 17039416 | 80254980 | QTL_ID=24883 | Body_weight_(336_days) | bw |
| chr4 | 17039416 | 80254980 | QTL_ID=24855 | Body_weight_(42_days) | bw |
| chr4 | 17039416 | 80254980 | QTL_ID=24890 | Body_weight_(504_days) | bw |
| chr4 | 17039416 | 80254980 | QTL_ID=24866 | Body_weight_(84_days) | bw |
| chr4 | 17039416 | 80254980 | QTL_ID=14457 | Body_weight_(day_of_f.intakerst_egg) | bw |
| chr4 | 17039416 | 80254980 | QTL_ID=14464 | Body_weight_(day_of_f.intakerst_egg) | bw |
| chr4 | 17039416 | 80254980 | QTL_ID=14470 | Body_weight_(day_of_f.intakerst_egg) | bw |
| chr4 | 30753891 | 88270681 | QTL_ID=55905 | Body_weight_(35_days) | bw |
| chr4 | 32166525 | 88270681 | QTL_ID=55930 | Growth_(0-35_days) | grow |
| chr4 | 46739707 | 88408499 | QTL_ID=2008 | Body_weight | bw |
| chr4 | 46739707 | 88408499 | QTL_ID=2015 | Body_weight | bw |
| chr4 | 46739707 | 88408499 | QTL_ID=2016 | Body_weight | bw |
| chr4 | 46739707 | 88408499 | QTL_ID=9759 | Body_weight_(42_days) | bw |
| chr4 | 46739707 | 88408499 | QTL_ID=9760 | Body_weight_(63_days) | bw |
| chr4 | 46739707 | 88408499 | QTL_ID=9761 | Growth_(21-42_days) | grow |
| chr4 | 46739707 | 88408499 | QTL_ID=9762 | Growth_(42-63_days) | grow |
| chr4 | 47497706 | 81608525 | QTL_ID=12498 | Growth_(0-14_days) | grow |
| chr4 | 47897157 | 84135947 | QTL_ID=12500 | Growth_(28-42_days) | grow |
| chr4 | 51678002 | 81608525 | QTL_ID=12499 | Growth_(14-28_days) | grow |
| chr4 | 61658027 | 87975245 | QTL_ID=12501 | Growth_(42-56_days) | grow |
| chr4 | 67602577 | 86114364 | QTL_ID=55942 | Growth_(105-140_days) | grow |
| chr4 | 68050486 | 84928400 | QTL_ID=17069 | Body_weight_(224_days) | bw |
| chr4 | 68567303 | 80565058 | QTL_ID=55938 | Growth_(70-105_days) | grow |
| chr4 | 69497574 | 81401798 | QTL_ID=55915 | Body_weight_(105_days) | bw |
| chr4 | 69942858 | 88408499 | QTL_ID=11766 | Body_weight_(35_days) | bw |
| chr4 | 69942858 | 84618310 | QTL_ID=7181 | Body_weight_(35_days) | bw |
| chr4 | 69942858 | 84618310 | ***QTL_ID=7157** | Body_weight_(35_days) | bw |
| chr4 | 69942858 | 84618310 | ***QTL_ID=7185** | Body_weight_(41_days) | bw |
| chr4 | 69942858 | 84618310 | ***QTL_ID=7162** | Body_weight_(41_days) | bw |
| chr4 | 69942858 | 88408499 | QTL_ID=11769 | Body_weight_(49_days) | bw |
| chr4 | 69942858 | 88408499 | QTL_ID=11773 | Body_weight_(63_days) | bw |
| chr4 | 70976493 | 80254980 | QTL_ID=2026 | Body_weight | bw |
| chr4 | 70976493 | 80254980 | QTL_ID=2023 | Feed_intake | f.intake |
| chr5 | 13971153 | 59580361 | QTL_ID=55939 | Growth_(70-105_days) | grow |
| chr5 | 22465118 | 58876488 | QTL_ID=17079 | Body_weight_(140_days) | bw |
| chr5 | 30162850 | 59580361 | QTL_ID=9763 | Growth_(42-63_days) | grow |
| chr6 | 1732132 | 29763072 | QTL_ID=55916 | Body_weight_(105_days) | bw |
| chr6 | 14060241 | 31613296 | QTL_ID=2125 | Body_weight | bw |
| chr6 | 26971183 | 31244082 | QTL_ID=24843 | Body_weight_(21_days) | bw |
| chr6 | 26971183 | 31244082 | QTL_ID=9764 | Body_weight_(42_days) | bw |
| chr6 | 26971183 | 31244082 | QTL_ID=24867 | Body_weight_(84_days) | bw |
| chr6 | 26971183 | 31244082 | QTL_ID=9765 | Growth_(21-42_days) | grow |
| chr6 | 28380699 | 29847153 | QTL_ID=12502 | Growth_(0-14_days) | grow |
| chr7 | 1293359 | 22946278 | QTL_ID=2136 | Body_weight | bw |
| chr7 | 1293359 | 27006229 | QTL_ID=2149 | Body_weight | bw |
| chr7 | 1293359 | 27006229 | QTL_ID=2151 | Body_weight | bw |
| chr7 | 1293359 | 27006229 | QTL_ID=2153 | Body_weight | bw |
| chr7 | 1293359 | 28666221 | QTL_ID=2147 | Body_weight | bw |
| chr7 | 1293359 | 28666221 | QTL_ID=2148 | Body_weight | bw |
| chr7 | 1293359 | 22946278 | QTL_ID=6626 | Body_weight_(112_days) | bw |
| chr7 | 1293359 | 22946278 | QTL_ID=6627 | Body_weight_(200_days) | bw |
| chr7 | 4794981 | 24245453 | QTL_ID=2150 | Body_weight | bw |
| chr7 | 7699967 | 36245040 | QTL_ID=2146 | Body_weight | bw |
| chr7 | 7699967 | 36245040 | QTL_ID=2158 | Body_weight | bw |
| chr7 | 7699967 | 36245040 | QTL_ID=2160 | Body_weight | bw |
| chr7 | 12980232 | 24245453 | QTL_ID=17305 | Body_weight_(14_days) | bw |
| chr7 | 12980232 | 24245453 | QTL_ID=17308 | Body_weight_(35_days) | bw |
| chr7 | 12980232 | 24245453 | QTL_ID=17314 | Body_weight_(77_days) | bw |
| chr7 | 24245453 | 28666221 | QTL_ID=17309 | Body_weight_(42_days) | bw |
| chr7 | 24245453 | 28666221 | QTL_ID=17310 | Body_weight_(49_days) | bw |
| chr7 | 24245453 | 28666221 | QTL_ID=17311 | Body_weight_(56_days) | bw |
| chr7 | 24245453 | 28666221 | QTL_ID=17312 | Body_weight_(63_days) | bw |
| chr7 | 24245453 | 28666221 | QTL_ID=17304 | Body_weight_(7_days) | bw |
| chr7 | 24245453 | 28666221 | QTL_ID=17313 | Body_weight_(70_days) | bw |
| chr7 | 24245453 | 28666221 | QTL_ID=17315 | Body_weight_(84_days) | bw |
| chr7 | 25665007 | 26638454 | QTL_ID=64529 | Feed_conversion_ratio | f.conv |
| chr7 | 25675920 | 26648497 | QTL_ID=95407 | Body_weight_(21_days) | bw |
| chr7 | 28666221 | 36245040 | QTL_ID=6625 | Body_weight_(8_days) | bw |
| chr7 | 34723350 | 36245040 | ***QTL_ID=7163** | Body_weight_(41_days) | bw |
| chr8 | 6726971 | 28767244 | QTL_ID=24891 | Body_weight_(504_days) | bw |
| chr8 | 6967382 | 28767244 | QTL_ID=55922 | Body_weight_(140_days) | bw |
| chr8 | 6967382 | 28767244 | QTL_ID=55940 | Growth_(70-105_days) | grow |
| chr8 | 8398681 | 28767244 | QTL_ID=2190 | Body_weight | bw |
| chr8 | 8398681 | 28767244 | QTL_ID=2199 | Body_weight | bw |
| chr8 | 8398681 | 28767244 | QTL_ID=2201 | Body_weight | bw |
| chr8 | 8398681 | 28767244 | QTL_ID=9767 | Body_weight_(63_days) | bw |
| chr8 | 8398681 | 28767244 | QTL_ID=9768 | Growth_(21-42_days) | grow |
| chr8 | 19901881 | 28767244 | QTL_ID=24877 | Body_weight_(168_days) | bw |
| chr8 | 19901881 | 28767244 | QTL_ID=24844 | Body_weight_(21_days) | bw |
| chr8 | 19901881 | 28767244 | QTL_ID=24857 | Body_weight_(42_days) | bw |
| chr8 | 19901881 | 28767244 | QTL_ID=24868 | Body_weight_(84_days) | bw |
| chr8 | 19901881 | 28767244 | QTL_ID=14458 | Body_weight_(day_of_f.intakerst_egg) | bw |
| chr8 | 19901881 | 28767244 | QTL_ID=14471 | Body_weight_(day_of_f.intakerst_egg) | bw |
| chr9 | 5124631 | 18131981 | QTL_ID=2217 | Body_weight | bw |
| chr9 | 6503007 | 23441680 | QTL_ID=9442 | Body_weight_(63_days) | bw |
| chr9 | 13658592 | 19669473 | QTL_ID=6634 | Body_weight_(200_days) | bw |
| chr9 | 13658592 | 23441680 | QTL_ID=24886 | Body_weight_(336_days) | bw |
| chr9 | 13658592 | 23441680 | QTL_ID=24869 | Body_weight_(84_days) | bw |
| chr9 | 13658592 | 19669473 | QTL_ID=6635 | Growth_(46-112_days) | grow |
| chr9 | 19669473 | 23441680 | ***QTL_ID=7177** | Body_weight_(1_day) | bw |
| chr10 | 1114728 | 15658282 | QTL_ID=55923 | Body_weight_(140_days) | bw |
| chr10 | 1924750 | 17334850 | QTL_ID=55907 | Body_weight_(35_days) | bw |
| chr10 | 2119755 | 17529855 | QTL_ID=55931 | Growth_(0-35_days) | grow |
| chr10 | 2142052 | 19911089 | QTL_ID=55911 | Body_weight_(70_days) | bw |
| chr10 | 3972100 | 17904865 | QTL_ID=55917 | Body_weight_(105_days) | bw |
| chr10 | 15658282 | 19911089 | QTL_ID=2234 | Body_weight_(ascites_conditions) | bw |
| chr10 | 16519830 | 19911089 | ***QTL_ID=7158** | Body_weight_(35_days) | bw |
| chr10 | 16519830 | 19911089 | ***QTL_ID=7164** | Body_weight_(41_days) | bw |
| chr11 | 888408 | 19401079 | QTL_ID=17080 | Body_weight_(140_days) | bw |
| chr11 | 975081 | 8298366 | QTL_ID=64559 | Feed_intake | f.intake |
| chr11 | 1063950 | 19185191 | QTL_ID=55924 | Body_weight_(140_days) | bw |
| chr11 | 2091236 | 3659428 | QTL_ID=24845 | Body_weight_(21_days) | bw |
| chr11 | 2091236 | 3659428 | QTL_ID=24858 | Body_weight_(42_days) | bw |
| chr13 | 721942 | 17760035 | QTL_ID=24859 | Body_weight_(42_days) | bw |
| chr13 | 721942 | 17760035 | QTL_ID=24870 | Body_weight_(84_days) | bw |
| chr13 | 721942 | 17760035 | QTL_ID=14472 | Body_weight_(day_of_f.intakerst_egg) | bw |
| chr13 | 8120084 | 17760035 | QTL_ID=2310 | Body_weight | bw |
| chr13 | 8120084 | 17760035 | QTL_ID=9769 | Body_weight_(21_days) | bw |
| chr13 | 8120084 | 17760035 | QTL_ID=9770 | Body_weight_(42_days) | bw |
| chr13 | 8120084 | 17760035 | QTL_ID=9771 | Body_weight_(63_days) | bw |
| chr13 | 8120084 | 17760035 | QTL_ID=9772 | Growth_(21-42_days) | grow |
| chr13 | 9323148 | 14111077 | QTL_ID=9444 | Body_weight_(63_days) | bw |
| chr13 | 10363506 | 16327806 | QTL_ID=6645 | Body_weight_(46_days) | bw |
| chr13 | 10363506 | 16327806 | QTL_ID=6646 | Growth_(1-8_days) | grow |
| chr13 | 12437243 | 17760035 | QTL_ID=17081 | Body_weight_(140_days) | bw |
| chr13 | 14111077 | 16327806 | QTL_ID=2304 | Body_weight | bw |
| chr14 | 50307 | 7445319 | QTL_ID=6647 | Body_weight_(1_day) | bw |
| chr14 | 50307 | 14696724 | QTL_ID=17082 | Body_weight_(140_days) | bw |
| chr14 | 50307 | 14696724 | QTL_ID=55943 | Growth_(105-140_days) | grow |
| chr14 | 133818 | 14696724 | QTL_ID=55925 | Body_weight_(140_days) | bw |
| chr14 | 3987816 | 7445319 | QTL_ID=2328 | Body_weight | bw |
| chr15 | 2798507 | 10631416 | QTL_ID=24887 | Body_weight_(336_days) | bw |
| chr15 | 3717446 | 8184057 | QTL_ID=3355 | Body_weight_(35_days) | bw |
| chr15 | 3717446 | 10631416 | QTL_ID=6648 | Body_weight_(46_days) | bw |
| chr15 | 3717446 | 10631416 | QTL_ID=6649 | Growth_(8-46_days) | grow |
| chr17 | 2449728 | 6121982 | QTL_ID=2355 | Body_weight | bw |
| chr18 | 1430572 | 4653744 | QTL_ID=6650 | Body_weight_(8_days) | bw |
| chr18 | 1430572 | 4653744 | QTL_ID=6651 | Growth_(1-8_days) | grow |
| chr18 | 3180498 | 7587685 | QTL_ID=9773 | Body_weight_(42_days) | bw |
| chr18 | 3180498 | 7587685 | QTL_ID=9774 | Growth_(21-42_days) | grow |
| chr21 | 13225 | 979078 | QTL_ID=95430 | Body_weight | bw |
| chr23 | 1779254 | 5097178 | QTL_ID=9124 | Growth_(post-challenge) | grow |
| chr26 | 58444 | 2538399 | QTL_ID=64571 | Feed_conversion_ratio | f.conv |
| chr26 | 1193077 | 4893906 | QTL_ID=9453 | Body_weight_(63_days) | bw |
| chr27 | 81131 | 4104720 | QTL_ID=55906 | Body_weight_(35_days) | bw |
| chr27 | 81131 | 4104720 | QTL_ID=55932 | Growth_(0-35_days) | grow |
| chr27 | 81131 | 4104720 | QTL_ID=55944 | Growth_(105-140_days) | grow |
| chr27 | 1210605 | 4104720 | QTL_ID=2410 | Body_weight | bw |
| chr27 | 1210605 | 4104720 | QTL_ID=2409 | Body_weight | bw |
| chr27 | 1969149 | 4104720 | QTL_ID=17084 | Body_weight_(140_days) | bw |
| chr27 | 2282360 | 4104720 | QTL_ID=55926 | Body_weight_(140_days) | bw |
| chr27 | 2467966 | 4104720 | QTL_ID=55912 | Body_weight_(70_days) | bw |
| chr27 | 2467966 | 4104720 | QTL_ID=55936 | Growth_(35-70_days) | grow |
| chr27 | 2595571 | 4104720 | QTL_ID=55918 | Body_weight_(105_days) | bw |
| chr27 | 3493381 | 4104720 | QTL_ID=2408 | Body_weight | bw |
| chr27 | 3493381 | 4104720 | ***QTL_ID=7159** | Body_weight_(35_days) | bw |
| chr28 | 1657793 | 4302316 | QTL_ID=24893 | Body_weight_(504_days) | bw |
| chr28 | 2073322 | 4302316 | QTL_ID=55901 | Body_weight_(hatch) | bw |
| chr28 | 2507636 | 4302316 | QTL_ID=2419 | Body_weight | bw |

*bolded are QTL mapped in the same F_2_ population used in this study

Supplementary Table S4.

| **Chr** | **Pos** | **Allele** | **#** | **Consequence** | **Ensemble gene ID** | **Gene Symbol** | **##** | **Overlapping QTLs** |
| --- | --- | --- | --- | --- | --- | --- | --- | --- |
| chr1 | 3706 | C/G | 1 | intron_variant | ENSGALG00000009771 |  |  | FALSE |
| chr1 | 25041791 | C/T | 2 | intergenic_variant |  |  |  | TRUE |
| chr1 | 45468342 | T/C | 3 | 3_prime_UTR_variant | ENSGALG00000011406 | *NTN4* |  | TRUE |
| chr1 | 53749693 | G/A | 4 | upstream_gene_variant | ENSGALG00000012647 | *RFX4* |  | TRUE |
| chr1 | 82575973 | T/C | 5 | downstream_gene_variant | ENSGALG00000027337 | *CLDND1* |  | TRUE |
| chr1 | 102944294 | A/C | 6 | intergenic_variant |  |  |  | TRUE |
| chr1 | 178184151 | G/A | 7 | intergenic_variant |  |  |  | TRUE |
| chr1 | 194726625 | A/C | 8 | intron_variant | ENSGALG00000017320 | *RAB6A* |  | TRUE |
| chr2 | 33436237 | A/T | 9 | intergenic_variant |  |  | 1 | TRUE |
| chr2 | 33437336 | C/G | 10 | intergenic_variant |  |  |  | TRUE |
| chr2 | 138815862 | G/A | 11 | intergenic_variant |  |  |  | TRUE |
| chr3 | 77435334 | C/T | 12 | intron_variant | ENSGALG00000015860 | *UBE3D* |  | TRUE |
| chr4 | 3514658 | G/T | 13 | intron_variant | ENSGALG00000029157 | *MBNL3* |  | TRUE |
| chr4 | 68623163 | A/C | 14 | intergenic_variant |  |  | 2 | TRUE |
| chr4 | 68882750 | G/A | 15 | 3_prime_UTR_variant | ENSGALG00000014320 | *UGDH* |  | TRUE |
| chr4 | 68882765 | T/G | 16 | downstream_gene_variant | ENSGALG00000014312 | *LIAS* |  | TRUE |
|  |  |  |  | 3_prime_UTR_variant | ENSGALG00000014320 | *UGDH* |  |  |
| chr4 | 69297525 | G/A | 17 | downstream_gene_variant | ENSGALG00000014312 | *LIAS* |  | TRUE |
| chr4 | 69370079 | A/T | 18 | intron_variant | ENSGALG00000013521 | *TBC1D1* |  | TRUE |
| chr4 | 69372005 | T/C | 19 | intron_variant | ENSGALG00000013521 | *TBC1D1* |  | TRUE |
| chr4 | 69372065 | G/A | 20 | intron_variant | ENSGALG00000013521 | *TBC1D1* |  | TRUE |
| chr4 | 73210325 | C/T | 21 | intergenic_variant |  |  | 3 | TRUE |
| chr4 | 73407243 | A/G | 22 | intergenic_variant |  |  |  | TRUE |
| chr4 | 73407249 | G/A | 23 | intergenic_variant |  |  |  | TRUE |
| chr4 | 75842191 | A/C | 24 | intron_variant | ENSGALG00000014485 | *LDB2* |  | TRUE |
| chr4 | 79707389 | C/G | 25 | intron_variant | ENSGALG00000028116 |  |  | TRUE |
| chr5 | 54401932 | C/T | 26 | intergenic_variant |  |  |  | TRUE |
| chr6 | 2997851 | G/A | 27 | intron_variant | ENSGALG00000002327 | *NRG3* |  | TRUE |
| chr6 | 5492179 | A/C | 28 | intergenic_variant |  |  |  | TRUE |
| chr6 | 28967516 | G/T | 29 | intergenic_variant |  |  |  | TRUE |
| chr6 | 31436207 | T/C | 30 | intergenic_variant |  |  |  | TRUE |
| chr7 | 6904443 | G/A | 31 | synonymous_variant |  |  |  | TRUE |
| chr7 | 22092999 | G/C | 32 | intron_variant | ENSGALG00000025739 | *RUFY4* | 4 | TRUE |
| chr7 | 22093009 | C/T | 33 | intron_variant | ENSGALG00000025739 | *RUFY4* |  | TRUE |
| chr7 | 26252164 | C/T | 34 | intergenic_variant |  |  |  | TRUE |
| chr7 | 34668134 | A/C | 35 | missense_variant | ENSGALG00000012484 | *RIF1* | 5 | TRUE |
| chr7 | 34855716 | G/A | 36 | upstream_gene_variant | ENSGALG00000012511 | *CACNB4* |  | TRUE |
| chr8 | 5848411 | T/C | 37 | intron_variant | ENSGALG00000003893 | *XPR1* |  | FALSE |
| chr8 | 27651130 | T/C | 38 | downstream_gene_variant | ENSGALG00000027967 | *MIR6630* |  | TRUE |
|  |  |  |  | upstream_gene_variant | ENSGALG00000011238 | *WLS* |  |  |
| chr9 | 15349595 | G/A | 39 | downstream_gene_variant | ENSGALG00000006246 |  |  | TRUE |
| chr9 | 21971358 | C/T | 40 | intron_variant | ENSGALG00000009669 | *RSRC1* |  | TRUE |
| chr10 | 9890328 | A/G | 41 | intron_variant | ENSGALG00000005011 | *SHC4* |  | TRUE |
| chr10 | 17086845 | G/A | 42 | intron_variant | ENSGALG00000026468 | *CHSY1* |  | TRUE |
| chr11 | 1249418 | G/A | 43 | missense_variant | ENSGALG00000117983 | *MUC5B* | 6 | TRUE |
| chr11 | 1997302 | G/A | 44 | intron_variant | ENSGALG00000002853 | *CFDP1* |  | TRUE |
| chr11 | 2149223 | A/G | 45 | intron_variant | ENSGALG00000003084 | *NUDT21* |  | TRUE |
|  |  |  |  | upstream_gene_variant | ENSGALG00000003071 | *OGFOD1* |  |  |
| chr13 | 1958055 | C/T | 46 | downstream_gene_variant | ENSGALG00000002447 | *CTNNA1* |  | TRUE |
| chr13 | 9894951 | C/T | 47 | downstream_gene_variant | ENSGALG00000002457 | *SIL1* | 7 | TRUE |
|  |  |  |  | intron_variant | ENSGALG00000003512 | *SPINK7* |  |  |
| chr13 | 9894952 | C/T | 48 | intron_variant | ENSGALG00000003512 | *SPINK7* |  | TRUE |
| chr13 | 12804655 | T/C | 49 | intergenic_variant |  |  |  | TRUE |
| chr13 | 15109350 | T/C | 50 | intron_variant | ENSGALG00000006424 | *JADE2* | 8 | TRUE |
| chr13 | 15109354 | C/T | 51 | intron_variant | ENSGALG00000006424 | *JADE2* |  | TRUE |
| chr14 | 2918142 | C/A | 52 | intron_variant | ENSGALG00000004224 | *MAD1L1* |  | TRUE |
| chr14 | 4019669 | G/T | 53 | intron_variant | ENSGALG00000004504 | *RADIL* |  | TRUE |
| chr14 | 12043802 | T/C | 54 | downstream_gene_variant | ENSGALG00000007456 | *CGTHBA* | 9 | TRUE |
|  |  |  |  | 3_prime_UTR_variant | ENSGALG00000028691 | *MPG* |  |  |
| chr14 | 12127914 | G/A | 55 | downstream_gene_variant | ENSGALG00000007473 | *MRPL28* |  | TRUE |
| chr14 | 12127934 | C/T | 56 | downstream_gene_variant | ENSGALG00000007473 | *MRPL28* |  | TRUE |
| chr15 | 6942668 | G/A | 57 | intergenic_variant |  |  |  | TRUE |
| chr15 | 9763685 | C/A | 58 | intergenic_variant |  |  |  | TRUE |
| chr17 | 1976385 | C/G | 59 | intron_variant | ENSGALG00000008475 | *ARRDC1* |  | FALSE |
| chr17 | 4917070 | C/G | 60 | intron_variant | ENSGALG00000005036 | *SH2D3C* | 10 | TRUE |
| chr17 | 4917071 | G/T | 61 | intron_variant | ENSGALG00000005036 | *SH2D3C* |  | TRUE |
| chr17 | 4917072 | T/G | 62 | intron_variant | ENSGALG00000005036 | *SH2D3C* |  | TRUE |
| chr17 | 5621341 | C/T | 63 | intergenic_variant |  |  | 11 | TRUE |
| chr17 | 5621374 | C/T | 64 | intergenic_variant |  |  |  | TRUE |
| chr17 | 5986188 | C/T | 65 | intergenic_variant |  |  |  | TRUE |
| chr18 | 4390068 | C/T | 66 | intron_variant | ENSGALG00000001971 | *UBE2O* |  | TRUE |
| chr18 | 9253828 | A/C | 67 | upstream_gene_variant | ENSGALG00000004429 | *COG1* | 12 | FALSE |
|  |  |  |  | downstream_gene_variant | ENSGALG00000004418 | *SS2R* |  |  |
|  |  |  |  | upstream_gene_variant | ENSGALG00000004429 | *COG1* |  |  |
| chr18 | 9551090 | C/T | 68 | intron_variant | ENSGALG00000006895 | *CEP131* |  | FALSE |
| chr18 | 9554600 | C/T | 69 | intron_variant | ENSGALG00000006895 | *CEP131* |  | FALSE |
| chr20 | 11847031 | T/C | 70 | intron_variant | ENSGALG00000007636 | *PEPCK* | 13 | FALSE |
| chr20 | 11847038 | C/T | 71 | intron_variant | ENSGALG00000007636 | *PEPCK* |  | FALSE |
| chr21 | 725260 | G/A | 72 | intergenic_variant |  |  |  | TRUE |
| chr23 | 2109713 | G/A | 73 | downstream_gene_variant | ENSGALG00000026379 | *OPRD1* |  | TRUE |
|  |  |  |  | downstream_gene_variant | ENSGALG00000021931 | *SNORA73* |  |  |
|  |  |  |  | downstream_gene_variant | ENSGALG00000002871 | *PHACTR4* |  |  |
|  |  |  |  | intron_variant | ENSGALG00000027784 | *RCC1* |  |  |
| chr23 | 4929597 | A/G | 74 | intron_variant | ENSGALG00000026836 | *COL16A1* |  | TRUE |
| chr25 | 601833 | T/C | 75 | intron_variant | ENSGALG00000014559 | *MEF2D* |  | FALSE |
| chr26 | 534090 | C/T | 76 | intron_variant | ENSGALG00000000427 | *KDM5B* | 14 | TRUE |
| chr26 | 1249195 | A/G | 77 | upstream_gene_variant | ENSGALG00000000329 | *AHCYL1* |  | TRUE |
| chr27 | 1587217 | C/A | 78 | 3_prime_UTR_variant | ENSGALG00000000201 | *PLEKHM1* | 15 | TRUE |
|  |  |  |  | upstream_gene_variant | ENSGALG00000025745 | *ARHGAP27* |  |  |
| chr27 | 1587218 | A/C | 79 | 3_prime_UTR_variant | ENSGALG00000000201 | *PLEKHM1* |  | TRUE |
|  |  |  |  | upstream_gene_variant | ENSGALG00000025745 | *ARHGAP27* |  |  |
| chr27 | 1587219 | G/A | 80 | 3_prime_UTR_variant | ENSGALG00000000201 | *PLEKHM1* |  | TRUE |
|  |  |  |  | upstream_gene_variant | ENSGALG00000025745 | *ARHGAP27* |  |  |
| chr27 | 1587220 | G/T | 81 | 3_prime_UTR_variant | ENSGALG00000000201 | *PLEKHM1* |  | TRUE |
|  |  |  |  | upstream_gene_variant | ENSGALG00000025745 | *ARHGAP27* |  |  |
| chr27 | 1587221 | G/C | 82 | 3_prime_UTR_variant | ENSGALG00000000201 | *PLEKHM1* |  | TRUE |
|  |  |  |  | upstream_gene_variant | ENSGALG00000025745 | *ARHGAP27* |  |  |
| chr27 | 1587222 | A/C | 83 | 3_prime_UTR_variant | ENSGALG00000000201 | *PLEKHM1* |  | TRUE |
|  |  |  |  | upstream_gene_variant | ENSGALG00000025745 | *ARHGAP27* |  |  |
| chr27 | 1587224 | A/C | 84 | 3_prime_UTR_variant | ENSGALG00000000201 | *PLEKHM1* |  | TRUE |
|  |  |  |  | upstream_gene_variant | ENSGALG00000025745 | *ARHGAP27* |  |  |
| chr27 | 1587226 | C/A | 85 | 3_prime_UTR_variant | ENSGALG00000000201 | *PLEKHM1* |  | TRUE |
|  |  |  |  | upstream_gene_variant | ENSGALG00000025745 | *ARHGAP27* |  |  |
| chr27 | 1587227 | C/G | 86 | 3_prime_UTR_variant | ENSGALG00000000201 | *PLEKHM1* |  | TRUE |
|  |  |  |  | upstream_gene_variant | ENSGALG00000025745 | *ARHGAP27* |  |  |
| chr27 | 1587228 | A/G | 87 | 3_prime_UTR_variant | ENSGALG00000000201 | *PLEKHM1* |  | TRUE |
|  |  |  |  | upstream_gene_variant | ENSGALG00000025745 | *ARHGAP27* |  |  |
| chr27 | 3798382 | C/G | 88 | intron_variant | ENSGALG00000027305 | *SKAP1* | 16 | TRUE |
| chr27 | 3798470 | G/A | 89 | intron_variant | ENSGALG00000027305 | *SKAP1* |  | TRUE |
| chr27 | 4784671 | G/A | 90 | intron_variant | ENSGALG00000003403 | *ZNF385C* |  | FALSE |
| chr28 | 788219 | C/T | 91 | downstream_gene_variant | ENSGALG00000027742 | *KLHL33* |  | FALSE |
| chr28 | 1252731 | G/A | 92 | intron_variant | ENSGALG00000026716 | *CELF5* |  | FALSE |
| chr28 | 2986566 | G/A |  | intergenic_variant |  |  |  | TRUE |
| chrZ | 9064309 | A/G | 94 | downstream_gene_variant | ENSGALG00000004022 | *TLN1* | 17 | FALSE |

# marker number; ##block number

Supplementary Table S5.

| **chr** | **start** | **end** | **QTL_ID** | **Trait** | **Abrev** |
| --- | --- | --- | --- | --- | --- |
| chr1 | 51977644 | 53798691 | ***QTL_ID=3324** | Body_weight_(35_days) | BW35 |
| chr1 | 53763599 | 53833783 | QTL_ID=19671 | Body_weight_(41_days) | BW41 |
| chr1 | 53763599 | 53833783 | QTL_ID=19672 | Feed_intake | F.intake |
| chr1 | 58033892 | 58104076 | QTL_ID=19675 | Body_weight_(41_days) | BW41 |
| chr1 | 58033892 | 58104076 | QTL_ID=19676 | Feed_intake | F.intake |
| chr1 | 83747978 | 90906992 | QTL_ID=12464 | Body_weight_(35_days) | BW35 |
| chr1 | 156472083 | 162032735 | QTL_ID=12469 | Body_weight_(35_days) | BW35 |
| chr2 | 15053040 | 19379651 | QTL_ID=7175 | Body_weight_(1_day) | BW1 |
| chr2 | 67519043 | 70985270 | QTL_ID=7170 | Body_weight_(35_days) | BW35 |
| chr2 | 79199747 | 101588000 | QTL_ID=7155 | Body_weight_(35_days) | BW35 |
| chr2 | 79199747 | 101588000 | QTL_ID=7160 | Body_weight_(41_days) | BW41 |
| chr2 | 79199747 | 101588000 | QTL_ID=7173 | Body_weight_(41_days) | BW41 |
| chr2 | 79199747 | 101588000 | QTL_ID=7179 | Body_weight_(35_days) | BW35 |
| chr2 | 79199747 | 101588000 | QTL_ID=7183 | Body_weight_(41_days) | BW41 |
| chr3 | 6645961 | 24160710 | ***QTL_ID=7184** | Body_weight_(41_days) | BW41 |
| chr3 | 6645961 | 106970113 | ***QTL_ID=7180** | Body_weight_(35_days) | BW35 |
| chr3 | 12229218 | 12229258 | QTL_ID=24377 | Body_weight_(35_days) | BW35 |
| chr3 | 12229218 | 12229258 | QTL_ID=24378 | Body_weight_(41_days) | BW41 |
| chr3 | 12229218 | 12229258 | QTL_ID=24379 | Body_weight_(42_days) | BW42 |
| chr3 | 24160710 | 35512024 | QTL_ID=7167 | Body_weight_(1_day) | BW1 |
| chr3 | 24160710 | 35512024 | QTL_ID=7171 | Body_weight_(35_days) | BW35 |
| chr3 | 24160710 | 35512024 | QTL_ID=7174 | Body_weight_(41_days) | BW41 |
| chr3 | 35512024 | 40606300 | QTL_ID=7156 | Body_weight_(35_days) | BW35 |
| chr3 | 35512024 | 40606300 | QTL_ID=7161 | Body_weight_(41_days) | BW41 |
| chr4 | 69942858 | 84618310 | ***QTL_ID=7157** | Body_weight_(35_days) | BW35 |
| chr4 | 69942858 | 84618310 | ***QTL_ID=7162** | Body_weight_(41_days) | BW41 |
| chr4 | 69942858 | 84618310 | ***QTL_ID=7185** | Body_weight_(41_days) | BW41 |
| chr6 | 29735045 | 31244082 | QTL_ID=7176 | Body_weight_(1_day) | BW1 |
| chr7 | 34723350 | 36245040 | ***QTL_ID=7163** | Body_weight_(41_days) | BW41 |
| chr9 | 19669473 | 23441680 | ***QTL_ID=7177** | Body_weight_(1_day) | BW1 |
| chr10 | 16519830 | 19911089 | ***QTL_ID=7158** | Body_weight_(35_days) | BW35 |
| chr10 | 16519830 | 19911089 | ***QTL_ID=7164** | Body_weight_(41_days) | BW41 |
| chr12 | 11106924 | 12275026 | QTL_ID=7168 | Body_weight_(1_day) | BW1 |
| chr18 | 378464 | 3180498 | QTL_ID=7169 | Body_weight_(1_day) | BW1 |
| chr18 | 378464 | 3180498 | QTL_ID=7172 | Body_weight_(35_days) | BW35 |
| chr18 | 378464 | 3180498 | QTL_ID=7182 | Body_weight_(35_days) | BW35 |
| chr27 | 1627441 | 3493381 | QTL_ID=7178 | Body_weight_(1_day) | BW1 |
| chr27 | 1627441 | 3493381 | QTL_ID=7186 | Body_weight_(41_days) | BW41 |
| chr27 | 3493381 | 4104720 | ***QTL_ID=7159** | Body_weight_(35_days) | BW35 |
| chr28 | 1674859 | 2912979 | QTL_ID=7187 | Body_weight_(41_days) | BW41 |

*bolded are QTLs overlapped by genome-wise associated and suggestively associated SNPs with the performance traits analyzed in this study

**References**

1. McLaren, W*. et a*l. Deriving the consequences of genomic variants with the Ensembl API and SNP Effect Predictor*. Bioinformati*c**s 2**6, 2069–70 (2010).
